# Homologous Recombination Repair Gene Mutation Characterization by Liquid Biopsy: A Phase II Trial of Olaparib and Abiraterone in Metastatic Castrate-Resistant Prostate Cancer

**DOI:** 10.3390/cancers13225830

**Published:** 2021-11-20

**Authors:** T. Hedley Carr, Carrie Adelman, Alan Barnicle, Iwanka Kozarewa, Sally Luke, Zhongwu Lai, Sally Hollis, Brian Dougherty, Elizabeth A. Harrington, Jinyu Kang, Fred Saad, Nuria Sala, Antoine Thiery-Vuillemin, Noel W. Clarke, Darren Hodgson, J. Carl Barrett

**Affiliations:** 1 AstraZeneca, Cambridge CB4 0WG, UK; alan.barnicle@astrazeneca.com (A.B.); iwanka.kozarewa@astrazeneca.com (I.K.); sally.luke@astrazeneca.com (S.L.); nigel.baker@astrazeneca.com (S.H.); liz.harrington@astrazeneca.com (E.A.H.); 2 AstraZeneca, Boston, MA 43183, USA; carrie.adelman@astrazeneca.com (C.A.); zhongwu.lai@astrazeneca.com (Z.L.); brian.dougherty@astrazeneca.com (B.D.); darren.hodgson@astrazeneca.com (D.H.); 3 AstraZeneca, Gaithersburg, MD 20878, USA; alice.kang@astrazeneca.com; 4 University of Montreal Hospital Research Centre, Montreal, QC H4A 3J1, Canada; fredsaad@videotron.ca; 5 Catalan Institute of Oncology, Hospital Josep Trueta, 17007 Girona, Spain; nsgonzalez@iconcologia.net; 6 Medical Oncology, CHU Besançon, 25000 Besançon, France; antoine.thieryvuillemin@oncologyfc2.onmicrosoft.com; 7 The Christie NHS Foundation Trust, Manchester M20 4BX, UK; Noel.Clarke@srft.nhs.uk

**Keywords:** circulating tumour DNA (ctDNA), homologous recombination repair (HRR), prostate cancer, next-generation sequencing (NGS), PARP inhibition, metastasis

## Abstract

**Simple Summary:**

Mutations in homologous recombination repair (HRR) genes are frequent in advanced prostate cancer and tumours harbouring these mutations have known sensitivity to PARP inhibitors, such as olaparib. In the randomized double-blind Phase II study (NCT01972218), olaparib and abiraterone prolonged radiographic progression-free survival (rPFS) versus placebo and abiraterone in patients with metastatic castration-resistant prostate cancer (mCRPC) unselected by HRR status. The study was designed to prioritize tumour samples for a pre-specified analysis of HRR status but was challenged by a low tissue submission rate. Circulating tumour DNA (ctDNA) and germline testing were initiated to supplement the assessment. Here, we present data from further germline and ctDNA analyses which increase the number of patients with confirmed HRR status. Our results support prior findings that patients with mCRPC benefit from olaparib and abiraterone treatment regardless of HRR status and highlight the value of ctDNA testing as a complement to tumour tissue sequencing.

**Abstract:**

Background: Phase III randomized trial data have confirmed the activity for olaparib in homologous recombination repair (HRR) mutated metastatic castration-resistant prostate cancer (mCRPC) post next-generation hormonal agent (NHA) progression. Preclinical data have suggested the potential for a combined effect between olaparib and NHAs irrespective of whether an HRR gene alteration was present. NCT01972217 was a randomised double-blind Phase II study which evaluated olaparib and abiraterone versus placebo and abiraterone in mCRPC patients who had received prior chemotherapy containing docetaxel. The study showed that radiologic progression was significantly delayed by the combination of olaparib and abiraterone regardless of homologous recombination repair mutation (HRRm) status. The study utilized tumour, blood (germline), and circulating tumour DNA (ctDNA) analysis to profile patient HRRm status, but tumour tissue provision was not mandated, leading to relatively low tissue acquisition and DNA sequencing success rates not representative of real-world testing. Patients and methods: Further analysis of germline and ctDNA samples has been performed for the trial to characterize HRRm status more fully and robustly analyse patient response to treatment. Results: Germline and plasma testing increased the HRRm characterized population from 27% to 68% of 142 randomized patients. Tumour-derived variants were detectable with high confidence in 78% of patients with a baseline plasma sample (71% of randomized patients). There was high concordance across methodologies (plasma vs. tumour; plasma vs. germline). The HR for the exploratory analysis of radiographic progression-free survival was 0.54 (95% CI: 0.32–0.93) in favour of olaparib and abiraterone in the updated HRR wild type (HRRwt) group (*n* = 73) and 0.62 (95% CI: 0.23–1.65) in the HRRm group (*n* = 23). Conclusion: Our results confirm the value of plasma testing for HRRm status when there is insufficient high-quality tissue for multi-gene molecular testing. We show that patients with mCRPC benefit from the combination of olaparib and abiraterone treatment regardless of HRRm status. The combination is currently being further investigated in the Phase III PROpel trial.

## 1. Introduction

Olaparib is a poly (adenosine diphosphate-ribose) polymerase (PARP) inhibitor (PARPi) used in the treatment of ovarian, breast, pancreatic, and prostate cancer [1,2,3,4,5,6]. PARP has a role in mediating repair of DNA single-strand breaks. Olaparib traps PARP on DNA, leading to double-strand breaks in cells undergoing DNA replication. In normal cells, these breaks are repaired via homologous recombination repair (HRR); however, in HRR-deficient cells, failure to repair these breaks results in cell death [7,8,9]. Monotherapy PARPi activity has been demonstrated in patients with advanced prostate cancer, including those that carry mutations in *BRCA1*, *BRCA2*, or other genes with direct or indirect roles in HRR that result in HRR deficiency [10,11,12,13]. Most recently, the Phase III PROfound study (NCT02987543) met its primary endpoint of a statistically significant and clinically meaningful improvement in radiographic progression-free survival (rPFS) and overall survival in patients with mCRPC who had a mutation in *BRCA1*, *BRCA2*, or *ATM* when they received olaparib. In an exploratory analysis, patients with mutations in 11 other genes with direct or indirect roles in HRR also benefited from olaparib (these 11 genes were selected based on mechanistic role in HRR, prevalence in key hereditary cancer indications, and preclinical and clinical evidence of PARPi sensitivity, and thus do not represent a definitive list of gene with direct or indirect roles in HRR) [5,6]. Based on this study, olaparib was approved in the USA for the treatment of metastatic castrate-resistant prostate cancer (mCRPC) patients with mutations in HRR genes [14].

Abiraterone is an anti-androgen agent used in the treatment of prostate cancer [15]. Evaluation of castration and androgen deprivation in prostate cancer has revealed a possible link between the androgen pathway and DNA damage response; inhibition of androgen signalling appears to reduce expression of HRR genes as well as the capacity for cells to repair DNA double-strand breaks, resulting in radiosensitivity and sensitization to PARP inhibition [16,17,18]. This suggests that patients treated with abiraterone may benefit from the addition of olaparib based on induction of HRR deficiency via non-genetic mechanisms. Additionally, PARP has been shown to facilitate expression of androgen receptor target genes, suggesting dual inhibition of androgen signalling as an additional cooperative mechanism between olaparib and abiraterone [19,20].

A Phase II study (NCT01972217) evaluating olaparib versus placebo in combination with abiraterone in patients with mCRPC who had received prior chemotherapy containing docetaxel was conducted to test the hypothesis that PARP inhibition plus next-generation anti-androgenic therapy would benefit patients regardless of HRR mutation (HRRm) status. The primary endpoint, investigator-assessed rPFS was significantly prolonged with olaparib plus abiraterone versus abiraterone alone in biomarker unselected patients (hazard ratio [HR] 0.65, 95% confidence interval [CI]: 0.44–0.97, *p* = 0.034) [21]. Unlike the later PROfound Phase III study, provision of an archival tumour sample or biopsy was not mandated by the protocol, nor were patients actively screened for eligible mutations in HRR genes. As a result, the number of tissue samples available for testing in this study was notably lower than in a real world setting and indeed that seen in PROfound.

In this study, to supplement the previous mutational analyses and increase our understanding of impact of HRRm status in this biomarker unselected population we performed additional post hoc germline sequencing and analysis of baseline plasma (circulating tumour DNA; ctDNA) samples. Further targeted next-generation sequencing (NGS) on plasma samples and low-pass whole genome (LPWG) analysis was undertaken to provide a more complete picture of HRRm status. This analysis increased the proportion of patients that could be robustly classified as being HRRm and at the same time greatly increased the proportion of patients which could be confidently labelled HRR wild type.

## 2. Materials and Methods

### 2.1. Study Design and Patients

This was a Phase II randomized, double-blind, placebo-controlled, multicentre study to compare olaparib versus placebo in addition to abiraterone treatment in patients with mCRPC who had received prior chemotherapy containing docetaxel (NCT01972217) [21]. Patients were randomized to receive olaparib tablets (standard dose: 300 mg twice daily) or placebo. All patients received abiraterone (1000 mg once daily) and prednisone or prednisolone (5 mg twice daily). This study was reported previously, alongside results based on initial analyses of tumour, blood, and plasma samples [21]. Study protocol and informed consent forms were subject to Ethics Committee or Institutional Review Board approval. Samples were collected following completion of appropriate written informed consent.

### 2.2. Tumour Sequencing

Formalin-fixed tumour samples (archival or fresh biopsy) were processed and sequenced by Foundation Medicine Inc. (FMI; Cambridge, MA, USA) using the Clinical Laboratory Improvement Amendments (CLIA) FoundationOne™ solid tumour test [22]. This test covers 15 genes with direct or indirect roles in HRR (BRCA1, BRCA2, ATM, BARD1, BRIP1, CDK12, CHEK1, CHEK2, FANCL, PALB2, PPP2R2A, RAD51B, RAD51C, RAD51D, and RAD54L).

### 2.3. Germline Sequencing

An optional blood sample for analysis of germline DNA was collected from patients who consented (*n* = 102). Blood was processed to DNA and banked according to established methods. Germline DNA was analysed via Color Genomics’ (Burlingame, CA, USA) CLIA 30-gene hereditary cancer sequencing test [23], which includes coverage of 9 of 15 HRR genes (BRCA1, BRCA2, ATM, BARD1, BRIP1, CHEK2, PALB2, RAD51C, RAD54L). Complete genome sequencing of the same DNA samples was facilitated via AstraZeneca’s Centre for Genomic Research, performed by Human Longevity Inc. (San Diego, CA, USA). The availability of whole genome data allowed the assessment of all 15 HRR genes and confirmation of variants seen in the prior test.

### 2.4. Circulating Tumour DNA Sequencing

Sequencing of circulating DNA (ctDNA) from plasma was undertaken both in-house and externally. Analysis of samples from patients with no tumour result, or a failed tumour result and no germline HRRm were prioritized (Supplemental Methods). For in-house analysis, whole-genome libraries were prepared and subjected to targeted sequencing using a 112-gene assay (AZ100; Table S1) utilizing custom xGen^®^ Lockdown^®^ probes (IDT, Leuven, Belgium). LPWG, when performed, utilized the same unselected libraries. Sequencing was performed using NextSeq500 or HiSeq4000 instruments (Illumina, Cambridge, UK). As part of separate efforts to understand emerging external assays, a subset of patients had baseline ctDNA sequenced using assays available from one or more commercial vendors including GuardantOMNI™ (Guardant Health, Redwood City, CA, USA) [24], FoundationACT (version 2) [25], and a customized liquid biopsy assay (ctDx HRR) provided by Resolution Bioscience Inc. (Kirkland, WA, USA) incorporating coverage of HRR genes of interest. The data processing and curation methods are described in the Supplemental Methods. 

### 2.5. HRR Subgroup Classification

Patients were classified into HRRm, HRR wild type (HRRwt), and HRR partially characterized subgroups as follows. HRRm patients were those harbouring at least one deleterious or suspected deleterious mutation detected by one or more of the test methods in any of the 15 HRR genes (variants of uncertain significance excluded); for patients with a large deletion in one of the HRR genes, clear evidence that this was biallelic loss was required. HRRwt comprised patients without a deleterious/suspected deleterious HRRm detected in tumour tissue and, or by, any other assay and patients with ctDNA fraction estimated to be ≥5% of total ctDNA with no HRRm detected in a plasma test (Supplemental Methods). HRR “partially characterized” patients were those without a tumour test result, who had no HRR gene mutation detected by germline and/or plasma test (where ctDNA fraction was <5% of total ctDNA) or were patients where all testing had failed or where samples were unavailable for analysis.

### 2.6. Statistical Methods

HRs and 95% CIs were calculated by log-rank test with the Breslow method for ties. Time-to-event endpoints were measured from randomization and medians and accompanying 95% CIs were calculated using the Kaplan–Meier method. All statistical analyses were conducted using SAS^®^ version 9.4 (SAS Institute, Cary, NC, USA). Reported *p*-values are two-sided, with *p*-values < 0.05 considered significant. Exploratory subgroup analyses of HRRm, HRRwt, and HRR partially characterized patients were predefined.

## 3. Results

### 3.1. Samples and Assays

**Figure 1:**
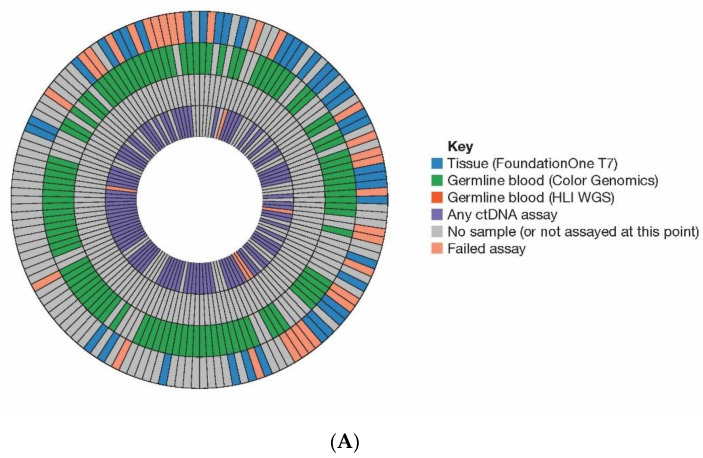

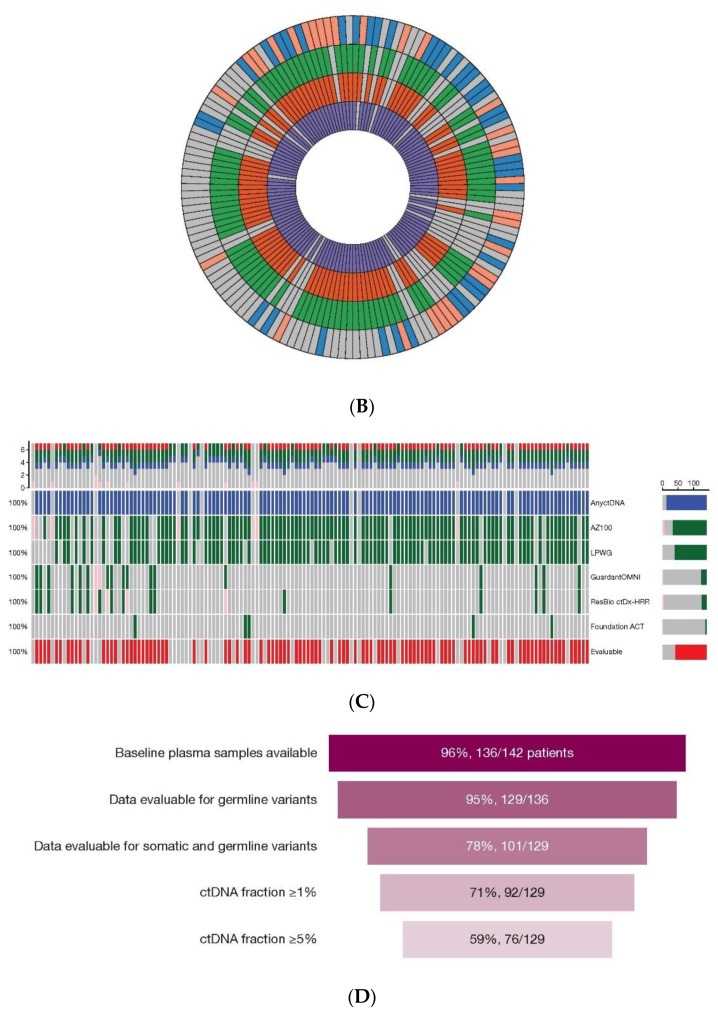
Summary of samples analysed. Illustration of the HRRm assays performed across tumour, germline and plasma (ctDNA) samples from patients in (**A**) initial and (**B**) final analyses for this study. Each segment represents a patient, and each tile within the segments represents the result from the relevant test. From the outer edge moving inwards, blue— tissue (FoundationOne), green—germline blood (Color Genomics), orange—germline blood (HLI WGS), purple—any ctDNA (plasma) assay. Pink/salmon indicates a failed test. Gray indicates no sample available or not assayed at that point. (**C**) Plasma assay results from the final analysis separated by specific assay. Patients were considered tumour shedders (“evaluable”) if there was high confidence detection of at least one somatic variant at or above 1% VAF not attributed to clonal haematopoiesis. (**D**) Outcomes of plasma sample analyses. Somatic alterations attributed to tumour or a clear signal of ctDNA were identified in 78% (101/129) of samples with a plasma test result. Of these 129 patients, analysis of ctDNA identified 76 (59%) with a tumour fraction ≥5%, 16 (12%) with a tumour fraction of 1–5%, and 9 (7%) had detectable tumour fraction at <1%. (ctDNA, circulating tumour DNA; HLI, Human Longevity Inc.; HRR, homologous recombination repair; HRRm, homologous recombination repair mutation; LPWG, low-pass whole genome; VAF, variant allele frequency; WGS, whole genome sequencing.).

We obtained HRR biomarker informative data from 136/142 (96%) randomized patients. Figure 1 illustrates where a tissue, blood (germline DNA), or baseline plasma (ctDNA) sample was analysed for each patient as part of the initial [21] (Figure 1A) or final analysis (Figure 1B) and assay success rate and missing samples are indicated. Figure 1C shows the distribution of assays applied to plasma only (final analysis). Full source data are available in Table S2. The HRR gene coverage of the assays used in this study is shown in Table 1.

### 3.2. Tumour Sequencing

Formalin-fixed, paraffin-embedded tumour samples for NGS were available for 68/142 (48%) patients. Of these, a result was reported for 38 patients (success rate 56%). For the 30 samples that failed, 23 (77%) failed on low DNA yield, 4 (13%) for low tumour cellularity, 2 (7%) due to an informatics QC metric fail, and 1 (3%) failed NGS library construction. Small biopsies with low tissue volumes represented the majority of the low DNA failures. From the 38 samples, three patients had deleterious variants in BRCA2, ATM, and CHEK2, respectively. The ATM and CHEK2 variants were confirmed as germline (Color Genomics data), whilst the BRCA2 variant was considered somatic (variant allele frequency [VAF] of 13%).

### 3.3. Germline Sequencing

A germline DNA sample was available from 102/142 (71%) patients. Sequencing was successful for all samples analysed via the Color Genomics assay. These data confirmed the germline ATM and CHEK2 variants identified in tumour samples and identified two additional ATM variants plus one additional deleterious variant in each of BRCA2, CHEK2, and PALB2 (all heterozygous) (Table S3). Assessment of tumour and germline data identified 8/142 (6.3%) patients with HRRm (germline and somatic combined). Whole genome sequencing (WGS) of these samples by Human Longevity Inc. (San Diego, CA, USA) confirmed the prior germline variants but did not identify any new HRRm variants. Classification of germline CHEK2 variants is described in the Supplemental Methods.

### 3.4. Plasma (ctDNA) Sequencing

Baseline plasma samples were available for 136/142 (96%) patients, of whom 96 were initially sequenced in-house using the AZ100 assay to provide data on HRRm status prior to Database Lock (DBL) (Figure 1C). Sequencing of the remaining samples was undertaken post-DBL for inclusion in the final dataset. Plasma volumes were variable and low (see Figure S1). There was no discernible site-specific pattern to the variability; instead, we attributed this to inconsistencies in collection and processing. Samples from 116 patients were processed for internal analysis and sufficient ctDNA obtained from 110 (95%) samples to support library generation, and targeted sequencing to provide information on (as a minimum) germline variants (see Materials and Methods). The median yield for in-house extractions was 9.6 ng/mL (standard deviation 50.7) (Table S4); median sequence coverage across all probes targeting the HRR genes for the AZ100 assay is shown in Figure S2. An additional 21 samples were analysed via GuardantOMNI™ and Resolution Bioscience ctDx HRR tests. Most (20/21) were from patients not already sequenced via the in-house assay and were almost completely overlapping (aliquots of the same plasma to both vendors). Five samples were sequenced using the in-house assay (AZ100) and the FoundationACT version 2 assay where residual plasma was available. Germline HRR status was ascertained in 95% (129/136) of patients for whom a plasma library was generated (Figure 1D). Somatic alterations and/or clear tumour signal were identified in 78% (101/129) of those samples, with 59% (76/129) demonstrating a tumour fraction ≥5%. These results should be considered conservative as many variants not meeting our quality criteria were excluded (Supplemental Methods). Compared with small variants, deletion events are considerably more difficult to detect with confidence in ctDNA assays, made more challenging in our samples due to variability in plasma volumes. We identified five patient samples with partial or whole gene deletion in one of the 15 HRR genes, one of which was a germline deletion in ATM. The other events were identified from ctDNA with a combination of AZ100 and LPWG. 

### 3.5. Assay Flow and HRRm Status

**Figure 2:**
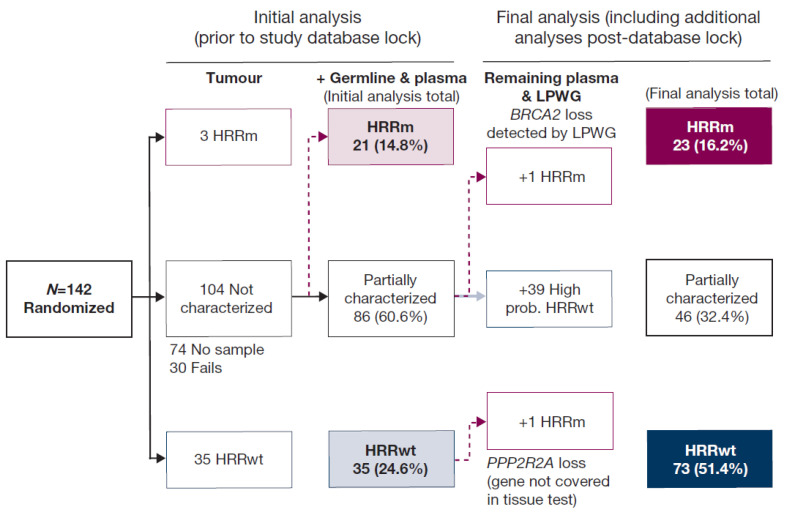
Assay flow and HRRm status. HRRm subgroup = patients with a HRRm detected by one or more of the tests. HRRwt subgroup (initial analysis) = patients without a HRRm detected in the tumour tissue test and no HRRm detected by any other assay. HRRwt subgroup (final analysis) = patients without a HRRm detected in the tumour tissue test and no HRRm detected by any other assay, and patients with ctDNA fraction estimated to be ≥5% of ctDNA, and no HRR gene mutation detected in a plasma test (these patients were considered high probability HRRwt due to ctDNA fraction ~10-fold higher than the limit of detection of the plasma assay, enabling robust detection of somatic variants). Partially characterized subgroup (initial analysis) = patients with no tissue test result, who had no HRRm detected by germline and/or plasma test, or patients where all testing failed, or no samples were available for analysis. Partially characterized subgroups (final analysis) = patients with no tissue test result, who had no HRRm detected by germline and/or plasma test (where ctDNA fraction was <5%), or patients where all testing failed, or no samples were available for analysis. (ctDNA, circulating tumour DNA; HRR, homologous recombination repair; HRRm, HRR mutation; HRRwt, HRR wild type; LPWG, low-pass whole genome; prob., probability).

From the initial analyses of tumour, germline, and plasma, 21 (15%) patients were identified as HRRm and 35 (25%) were defined as HRRwt. The remaining 86 (61%) patients were considered partially characterized (Figure 2). In the final analysis, two additional patients were classed as HRRm, giving a total of 23 (16%). The first of these events was a deep (homozygous) loss of BRCA2, identified in LPWG data from ctDNA and confirmed by the lack of evidence of loss of heterozygosity in the single-nucleotide polymorphisms covered by the AZ100 targeted assay (patient #99, visualized in Figure S3). The second was a homozygous loss of PPP2R2A detected in both targeted and LPWG ctDNA data (gene not covered by FMI T7 tissue test previously performed for this patient). Subsequent to the analyses described in this report, AstraZeneca’s internal evaluation of sensitivity in vitro and exploratory analysis of survival in the PROfound study indicated that PPP2R2A loss confers no sensitivity to PARPi therapy or overall survival benefit for patients on olaparib monotherapy (Supplemental Appendix [6]). We have retained this gene in our analyses here in order to maintain consistency with the primary study publication and to report the study as designed.

For the final analysis, 73 (51%) patients were classified as HRRwt based on additional data from ctDNA analysis. The final “partially characterized” subgroup comprised 46 (32.4%) patients.

### 3.6. HRRm Variants

**Figure 3:**
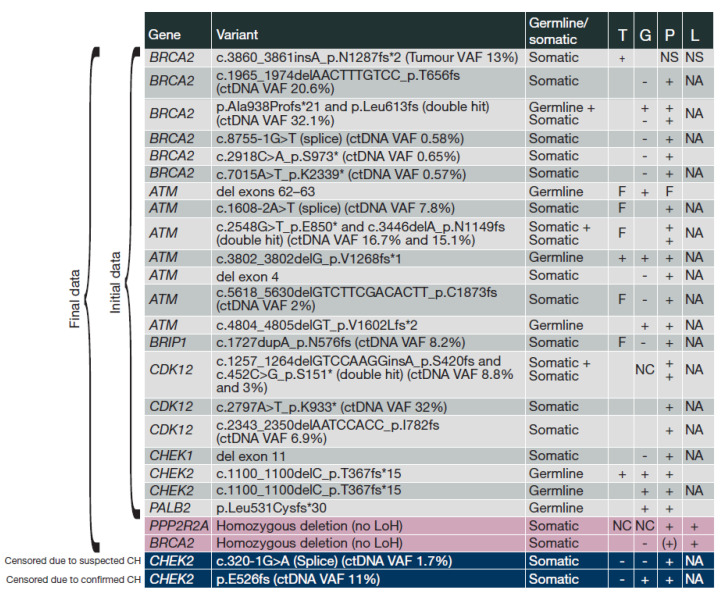
Summary of HRRm variants detected across all test modalities. One row per patient. VAF is shown for somatic variants (detected in tumour or ctDNA, and confirmed not present in germline, OR suspected somatic based on observed VAF). (CH, clonal haematopoiesis; ctDNA, circulating tumour DNA; F, test fail; G, germline test; HRRm, homologous recombination repair mutation; L, plasma low-pass whole genome; NA, not applicable (variant not detectable by technique); NC, gene not covered; NS, non-shedder; P, plasma-targeted next-generation sequencing test; T, tumour tissue test; VAF, variant allele frequency; +, mutation detected; –, mutation not detected; (+), mutation detected upon manual review of data based on signal in low-pass whole genome; Blank, no sample).

Data on HRRm detected across all test modalities are shown in Figure 3. Our additional analyses post-DBL added two HRRm patients beyond those previously reported [21]. Twenty-six mutations were observed in 23 patients (three had a double hit in BRCA2, ATM, or CDK12, respectively). Six (4%) patients had only germline HRRm, 16 (11%) had purely somatic HRRm, and one had both germline and somatic BRCA2 mutations. Of the 23 patients with HRRm, 15 (65%) were identified by a plasma result alone. BRCA2 and ATM mutations were most common, followed by CDK12 mutations; two patients had the same pathogenic germline CHEK2 variant (Supplemental Material—Classification of Germline CHEK2 Variants). The eight deleterious BRCA2 mutations (in seven patients) and eight ATM mutations (in seven patients) comprised three changes leading to a nonsense (Stop) codon, eight insertions or deletions leading to a frameshift, two base changes impacting canonical splice acceptor or donor sequences, and three partial or whole gene deletions. Due to the small number of patients carrying mutations in any single HRR gene, no single gene analyses were possible, based on the minimum threshold of five progression events per arm per subgroup as specified by the protocol. Hence, only aggregate HRR subgroup analyses were performed. In contrast, exploratory single-gene analyses were reported for a number of genes meeting these criteria in the monotherapy PROfound study [5], and future studies including PROpel will enable evaluation of individual genes in the context of the combination.

### 3.7. HRRm Concordance

Concordance for HRRm between tissue versus plasma samples and germline versus plasma samples was evaluated where overlapping data were available (Table S5). The single missed HRRm was a BRCA2 insertion (frameshift) mutation detected at 13% VAF in tumour but not observed in baseline plasma. No other high-confidence somatic mutations were seen in this sample, nor any evidence of tumour signal in LPWG data. This case was thus considered likely an example of a patient with low or non-detectable ctDNA at baseline (with available techniques). Overall, 28/129 (22%) of plasma samples that yielded sequence data from any plasma assay showed no variants that could be confidently described as likely of tumour origin.

### 3.8. Sensitivity Analysis for rPFS

**Figure 4:**
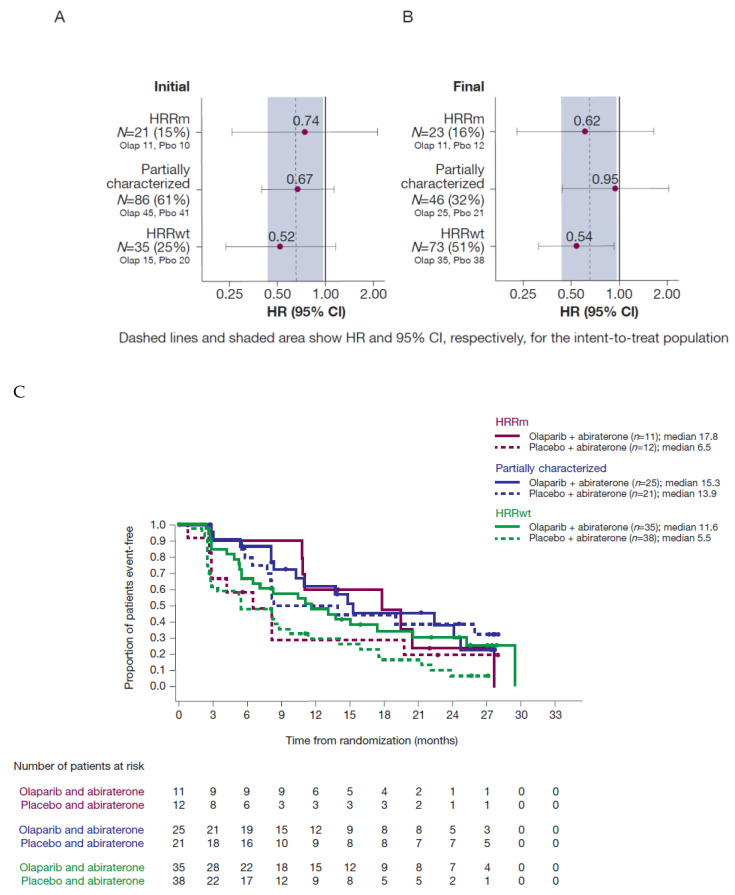
Forest plots by HRRm subgroup as defined in the (**A**) initial and (**B**) final datasets; dashed lines and shaded area show HR and 95% CI of the primary rPFS analysis, respectively, for the ITT population. Kaplan–Meier curves for the (**C**) HRRm, partially characterized and HRRwt subgroups in the final dataset. (CI, confidence interval; ctDNA, circulating tumour DNA; HR, hazard ratio; HRR, homologous recombination repair; HRRm, HRR mutation; HRRwt, HRR wild type; ITT, intent-to-treat; olap, olaparib; pbo, placebo; rPFS, radiographic progression-free survival; VAF, variant allele frequency).

With the more complete final analysis dataset, an updated sensitivity analysis of rPFS by HRR subgroup showed a similar effect in the HRRm and HRRwt groups to previously reported analyses, demonstrating consistent benefit from the combination irrespective of biomarker status (Figure 4 and Discussion). 

## 4. Discussion

Knowledge of HRR status is important in patients with mCRPC, as evidenced by the recent approvals of olaparib for the treatment of patients with HRR gene-mutated mCRPC and rucaparib for BRCA gene-mutated mCRPC [7,18,26]. Not all mCRPC patients may have enough tumour tissue or tissue of sufficient quality available to support multi-gene molecular testing. In this study, provision of tumour was not mandated, resulting in a relatively low acquisition rate for samples and a higher fail rate than expected in a real-world situation. With a more intensive collection protocol, as in the recent PROfound trial, a success rate of almost 70% is possible [5,27], and higher rates may be achieved in clinical practice [28]. Nevertheless, this still leaves a notable shortfall in the fraction of patients which could be characterized for HRRm. This could be partially filled by easily collected blood sampling and subsequent ctDNA analysis.

Analysis of ctDNA from mCRPC patients in this Phase II study increased the proportion of patients who could be assessed for HRR status from 30% (based on available tissue and germline samples) to 68% (once plasma was evaluated). Plasma analysis (interim and final combined) led to a near three-fold increase in the proportion of patients identified as HRRm, and a more than two-fold increase in the proportion of patients classified as HRRwt. The relatively high ctDNA fraction in mCRPC patients enabled robust detection of somatic tumour variants and supported high confidence classification of HRRwt cases. High concordance for HRRm status was also demonstrated where overlapping data were available for tissue versus plasma and germline versus plasma. 

We assigned a conservative requirement of 5% apparent tumour fraction with no HRRm detected to allow the designation of a patient as HRRwt based on plasma testing. The definition of HRRwt in patients where only a plasma result is available hinges on the sensitivity and specificity of assays to a variety of mutation types that may impact HRR genes. We have high confidence in our ability to detect point mutations and small indels down to 0.5% VAF with high specificity using our methods as described, and similarly high confidence with commercial vendor assays.

Larger-scale partial or whole gene deletions and rearrangements are of significance in mCRPC in some HRR genes (e.g., ~20% of BRCAm [29]) but, compared with small variants, such mutational events are considerably more difficult to detect with confidence in ctDNA-based assays. An additional challenge specific to this study was the variable and often low volumes of plasma available for ctDNA analysis. However, yields of ctDNA were sufficient to allow successful library generation in most cases, and sequencing of ctDNA was highly successful. We observed four whole or partial deep gene deletions from 76 patients with a successful plasma result and apparent ctDNA fraction ≥5% (giving a prevalence for deletions of 5% in this subset). This is comparable with publicly available tissue datasets where deep deletions across the 14 HRR genes (excluding PPP2R2A) are reported in 8% of mCRPC cases [30]. Based on these challenges, we cannot exclude the possibility our HRRwt, or partially characterized subgroups may include some patients with a deep deletion in one of the HRR genes, though the potential numbers of false-negative results of this type would be expected to be small. Whilst analysis of ctDNA at baseline in patients with mCRPC proved of high value in this study, it is acknowledged that the broader utility of ctDNA analyses is highly dependent on the disease burden and level of ctDNA shedding ongoing at the time of plasma sampling and such differences may lead to bias in the pickup/reporting of mutations in patients. Patients with progressive disease will clearly provide samples with more abundant (and more easily detected) ctDNA fraction, whilst patients on an effective systemic therapy may show low or non-detectable ctDNA levels. 

With the final biomarker data, an updated sensitivity analysis of rPFS by HRR subgroup showed a similar effect in the HRRm and HRRwt groups to that in previously reported analyses [21]. As shown in Figure 4, the estimated HR for the HRRwt group was virtually unchanged, despite the HRRwt subgroup more than doubling in size. The HRRm subgroup size increased only slightly with a modest effect on the estimated HR in favour of the combination therapy. The estimated HR for the now much smaller HRR partially characterized group moved from 0.67 (95% CI: 0.40–1.13) to 0.95 (95% CI: 0.44–2.04). Whilst the HR remained just within the 95% CI for the intent-to-treat population, the lower number of patients and events in this subgroup may have contributed to greater uncertainty in the estimates. Due to the small number of patients carrying mutations in any single HRR gene, no single-gene analyses were possible in NCT01972218. However, exploratory single-gene analyses were reported for a number of genes meeting the predefined statistical criteria in the PROfound study [5]. These showed the greatest benefit for the monotherapy olaparib treatment in the BRCAm subgroup, though several other genes also showed a positive trend. Future studies, including PROpel, will enable evaluation of individual genes in the context of the combination, which will be informative alongside the aggregate HRR subgroup data from both PROpel and this study. 

## 5. Conclusions

This study reinforces the value of plasma testing in cases where sufficient high-quality tumour tissue for advanced genomic profiling via NGS is not available. By increasing the number of patients that could be characterized for HRRm through plasma testing, we provide a more robust assessment of treatment efficacy in patients with and without HRRm. Our results are consistent with prior findings that mCRPC patients benefit from a combination of olaparib and abiraterone treatment regardless of HRRm status. The combination is currently being investigated in the Phase III randomized controlled PROpel trial of olaparib and abiraterone versus placebo and abiraterone in a first-line mCRPC setting.

## Figures and Tables

**Table 1:** Coverage of 15 HRR genes by the assays used in this study.

	*BRCA1*	*BRCA2*	*ATM*	*BARD1*	*BRIP1*	*CDK12*	*CHEK1*	*CHEK2*	*FANCL*	*PALB2*	*PPP2R2A*	*RAD51B*	*RAD51C*	*RAD51D*	*RAD54L*
**Initial analysis**															
FoundationOne (tumour tissue, CLIA)															
Color Genomics (blood, germline DNA, CLIA)															
AZ100 (plasma, ctDNA, RUO)	#	#	#	#	#	#	#	*	#	#	#	#	#	#	#
**Final analysis**															
Guardant OMNI (plasma, ctDNA, RUO)								*							
FoundationACT (v2) (plasma, ctDNA, CLIA)								*							
Resolution Bioscience custom ctDx HRR (plasma, ctDNA, RUO)								*							
AZ LPWG (plasma, ctDNA, RUO)	+	+	+	+	+	+	+	+	+	+	+	+	+	+	+
30× WGS (HLI, RUO)															

Gray blocks indicate genes covered by the assay, white-not covered. Assays covered entire coding sequences of all listed genes. Sensitivity for small mutations was typically 0.25–0.5% for all plasma ctDNA assays employed. * Some deleterious CHEK2 variants in ctDNA excluded from analysis due to confirmed/suspected clonal haematopoiesis of indeterminate potential. # Marked ctDNA assays also covered deletion/loss (sensitivity dependent on ctDNA input and tumour fraction). + LPWG plasma data allowed analysis of ctDNA copy number alterations only. The 30× WGS from blood allowed analysis for all variant types in germline. AZ, AstraZeneca; CLIA, clinical laboratory improvement amendments; ctDNA, circulating tumour DNA; HLI, Human Longevity Inc.; HRR, homologous recombination repair; LPWG, low-pass whole genome; RUO, research use only; WGS, whole genome sequencing.

## Data Availability

Data underlying the findings described in this manuscript may be obtained in accordance with AstraZeneca’s data sharing policy described at https://astrazenecagrouptrials.pharmacm.com/ST/Submission/Disclosure (accessed on 20 October 2021).

## Supplementary Materials

The following are available online at https://www.mdpi.com/article/10.3390/cancers13225830/s1, Figure S1: Plots illustrating (A) mass of ctDNA, and (B) volumes of plasma obtained per 2 × 1 mL aliquots of plasma provided by clinical site, Figure S2: Median unique read coverage of key HRR genes across all samples analyzed via AZ100 assay, Figure S3: Visualization of LPWG data from ctDNA libraries, Table S1: AZ100 gene list, hg38 gene coordinates and reference transcripts, Table S2: Pass and fail results for all samples across all assays performed at (A) initial analysis and (B) final analysis, Table S3: All HRRm mutations, Table S4: Metrics for all plasma samples analyzed in-house, Table S5: HRRm concordance between (A) tissue vs. plasma, and (B) germline vs. plasma.

## References

[1] Robson M., Im S.A., Senkus E., Xu B., Domcheck S.M., Masuda N., Delaloge M.D., Li W., Tung N., Armstrong A. (2017). Olaparib for metastatic breast cancer in patients with a germline BRCA mutation. N. Engl. J. Med..

[2] Golan T., Hammel P., Reni M., Van Cutsem E., Macarulla T., Hall M.J., Park J., Hochhauser D., Arnold D., Oh D. (2019). Maintenance olaparib for germline BRCA-mutated metastatic pancreatic cancer. N. Engl. J. Med..

[3] Moore K., Colombo N., Scambia G., Kim B., Oaknin A., Friedlander M., Lisyanskaya A., Floquet A., Leary A., Sonke G.S. (2018). Maintenance olaparib in patients with newly diagnosed advanced ovarian cancer. N. Engl. J. Med..

[4] Kaufman B., Shapira-Frommer R., Schmutzler R.K., Audeh M.W., Friedlander M., Balmana J., Mitchell G., Fried G., Stemmer S.M., Hubert A. (2015). Olaparib monotherapy in patients with advanced cancer and a germline BRCA1/2 mutation. J. Clin. Oncol..

[5] de Bono J., Mateo J., Fizazi K., Saad F., Shore N., Sandhu S., Chi K.N., Sartor O., Agarwal N., Olmos D. (2020). Olaparib for metastatic castration-resistant prostate cancer. N. Engl. J. Med..

[6] Hussain M., Mateo J., Fizazi K., Saad F., Shore N., Sandhu S., Chi K.N., Sartor O., Agarwal N., Olmos D. (2020). Survival with olaparib in metastatic castration-resistant prostate cancer. N. Engl. J. Med..

[7] O’Connor M.J. (2015). Targeting the DNA damage response in cancer. Mol. Cell.

[8] Farmer H., McCabe N., Lord C.J., Tutt A.N.J., Johnson D.A., Richardson T.B., Santarosa M., Dillon K.J., Hickson I., Knights C. (2005). Targeting the DNA repair defect in BRCA mutant cells as a therapeutic strategy. Nature.

[9] Pommier Y., O’Connor M.J., de Bono J. (2016). Laying a trap to kill cancer cells: PARP inhibitors and their mechanisms of action. Sci. Transl. Med..

[10] Abida W., Bryce A.H., Vogelzang N.J., Amato R.J., Percent I., Shapiro J.D., McDermott R., Hussain A., Patnaik A., Petrylak D. (2018). Preliminary results from TRITON2: A phase 2 study of rucaparib in patients (pts) with metastatic castration-resistant prostate cancer (MCRPC) associated with homologous recombination repair (HRR) gene alterations. Ann. Oncol..

[11] Mateo J., Porta N., McGovern U.B., Elliott T., Jones R.J., Syndikus I., Ralph C., Jain S., Varughese M.A., Parikh O. (2019). TOPARP-B: A phase II randomized trial of the poly(ADP)-ribose polymerase (PARP) inhibitor olaparib for metastatic castration resistant prostate cancers (mCRPC) with DNA damage repair (DDR) alterations. J. Clin. Oncol..

[12] Ryan C.J., Abida W., Bryce A.H., Balar A.V., Dumbadze I., Given R.W., Morris D., Petrylak D.P., Redfern C.H., Scher H.I. (2018). TRITON3: An international, randomized, open-label, phase III study of the PARP inhibitor rucaparib vs. physician’s choice of therapy for patients with metastatic castration-resistant prostate cancer (mCRPC) associated with homologous recombination deficiency (HRD). J. Clin. Oncol..

[13] Smith M.R., Sandhu S.K., Kelly W.K., Scher H.I., Efstathiou E., Lara P., Yu E.Y., George D.J., Chi K.N., Summa J. (2019). Phase II study of niraparib in patients with metastatic castration-resistant prostate cancer (mCRPC) and biallelic DNA-repair gene defects (DRD): Preliminary results of GALAHAD. J. Clin. Oncol..

[14] US Food and Drug Administration (FDA) LYNPARZA™ (Olaparib) Prescribing Information. https://www.accessdata.fda.gov/drugsatfda_docs/label/2020/208558s014lbl.pdf.

[15] De Bono J.S., Logothetis C.J., Molina A., Fizazi K., North S., Chu L., Chi K.N., Jones R.J., Goodman O.B., Saad F. (2011). Abiraterone and increased survival in metastatic prostate cancer. N. Engl. J. Med..

[16] Asim M., Tarish F., Zecchini H.I., Sanjiv K., Gelali E., Massie C.E., Baridi A., Warren A.Y., Zhao W., Ogris C. (2017). Synthetic lethality between androgen receptor signalling and the PARP pathway in prostate cancer. Nat. Commun..

[17] Polkinghorn W.R., Parker J.S., Lee M.X., Kass E.M., Spratt D.E., Iaquinta P.J., Arora V.K., Yen W.F., Cai L., Zheng D. (2013). Androgen receptor signaling regulates DNA repair in prostate cancers. Cancer Discov..

[18] Tarish F.L., Schultz N., Tanoglidi A., Hamberg H., Letocha H., Karaszi K., Hamdy F.C., Granfors T., Helleday T. (2015). Castration radiosensitizes prostate cancer tissue by impairing DNA double-strand break repair. Sci. Transl. Med..

[19] Ju B.G., Lunyak V.V., Perissi V., Garcia-Bassets I., Rose D.W., Glass C.K., Rosenfeld M.G. (2006). A topoisomerase IIbeta-mediated dsDNA break required for regulated transcription. Science.

[20] Schiewer M.J., Goodwin J.F., Han S., Brenner J.C., Augello M.A., Dean J.L., Liu F., Planck J.L., Ravindranathan P., Chinnaiyan A.M. (2012). Dual roles of PARP-1 promote cancer growth and progression. Cancer Discov..

[21] Clarke N., Wiechno P., Alekseev B., Sala N., Jones R., Kocak I., Chiuri V.E., Jassem J., Flechon A., Redfern C. (2018). Olaparib combined with abiraterone in patients with metastatic castration-resistant prostate cancer: A randomised, double-blind, placebo-controlled, phase 2 trial. Lancet Oncol..

[22] Frampton G.M., Fichtenholtz A., Otto G.A., Wang K., Downing S.R., He J., Schnall-Levin M., White J., Sanford E.M., An P. (2013). Development and validation of a clinical cancer genomic profiling test based on massively parallel DNA sequencing. Nat. Biotechnol..

[23] Neben C.L., Zimmer A.D., Stedden W., van den Akker J., O’Connor R., Chan R.C., Chen E., Tan Z., Leon A., Ji J. (2019). Multi-gene panel testing of 23,179 individuals for hereditary cancer risk identifies pathogenic variant carriers missed by current genetic testing guidelines. J. Mol. Diagn..

[24] Helman E., Artieri C., Vowles J.V., Yen J., Nance T., Sikora M., Gourneau J., Goel M., Mortimer S., Chudova D. (2018). Analytical validation of a comprehensive 500-gene ctDNA panel designed for immuno-oncology and DNA damage research. Proceedings of the AACR Annual Meeting 2018.

[25] Clark T.A., Chung J.H., Kennedy M., Hughes J.D., Chennagiri N., Lieber D.S., Fendler B., Young L., Zhao M., Coyne M. (2018). Analytical validation of a hybrid capture-based next-generation sequencing clinical assay for genomic profiling of cell-free circulating tumor DNA. J. Mol. Diagn..

[26] US Food and Drug Administration (FDA) RUBRACA™ (Rucaparib) Prescribing Information. https://www.accessdata.fda.gov/drugsatfda_docs/label/2020/209115s004lbl.pdf.

[27] Hussain M.H.A., Mateo J., Sandhu S.K., Fizazi K., Saad F., Shore N.D., Olmos D., Corcoran C., Sibilla C., Kohlmann A. (2020). Next-generation sequencing (NGS) of tumor tissue from >4000 men with metastatic castration-resistant prostate cancer (mCRPC): The PROfound phase III study experience. J. Clin. Oncol..

[28] Mateo J., McKay R., Abida W., Aggarwal R., Alumkal J., Alva A., Feng F., Gao X., Graff J., Hussain M. (2020). Accelerating precision medicine in metastatic prostate cancer. Nat. Cancer.

[29] Mateo J., Seed G., Bertan C., Rescigno P., Dolling D., Figueiredo I., Miranda S., Nava Rodrigues D., Gurel B., Clarke M. (2020). Genomics of lethal prostate cancer at diagnosis and castration resistance. J. Clin. Investig..

[30] Nguyen B., Mota J.M., Nandakumar S., Stopsack K.H., Weg E., Rathkopf D., Morris M.J., Scher H.I., Kantoff P.W., Gopalan A. (2020). Pan-cancer analysis of CDK12 alterations identifies a subset of prostate cancers with distinct genomic and clinical characteristics. Eur. Urol..

