# Supplementary material for: Homologous Recombination Repair Gene Mutation Characterization by Liquid Biopsy: A Phase II Trial of Olaparib and Abiraterone in Metastatic Castrate-Resistant Prostate Cancer
Source: Cancers (Basel). 2021 Nov 20;13(22):5830. doi: 10.3390/cancers13225830 (PMC8616430; doi:10.3390/cancers13225830)
Supplement: Supplementary file 1 [file cancers-13-05830-s001.zip › cancers-1449920-supplementary.pdf]

SUPPLEMENTAL APPENDIX

This appendix has been provided by the authors to give readers additional information about their work.

Supplement to: Carr TH, Adelman C, Barnicle A *et al*.

Homologous Recombination Repair Gene Mutation Characterization By Liquid Biopsy: A Phase II Trial of Olaparib and Abiraterone in Metastatic Castrate-Resistant Prostate Cancer

CONTENTS

SUPPLEMENTAL METHODS **3**

Sequence of Analyses of Patient Samples 3

Circulating Tumor DNA (ctDNA) Extraction/Purification 3

ctDNA Whole Genome Library Preparation 4

In-House Sequencing of ctDNA 4

Data Processing, Curation, and Visualization 4

Criteria for Homologous Recombination Repair Wild Type (HRRwt) Classification 6

Clonal hematopoiesis and *CHEK2* 6

Classification of Germline *CHEK2* Variants 6

SUPPLEMENTAL RESULTS **8**

Table S1. AZ100 gene list, hg38 gene coordinates and reference transcripts 8

Table S2. Pass and fail results for all samples across all assays performed at (A) initial analysis and (B) final analysis 12

Table S3. All HRRm mutations 34

Table S4. Metrics for all plasma samples analyzed in-house 36

Table S5. HRRm concordance between (A) tissue vs plasma, and (B) germline vs plasma 42

Figure S1. Plots illustrating (A) mass of ctDNA, and (B) volumes of plasma obtained per 2 × 1 mL aliquots of plasma provided by clinical site 43

Figure S2. Median unique read coverage of key HRR genes across all samples analyzed via AZ100 assay 44

Figure S3. Visualization of LPWG data from ctDNA libraries. 45

SUPPLEMENTAL REFERENCES **47**

SUPPLEMENTAL METHODS

Sequence of Analyses of Patient Samples

During the course of the study tumor samples (non-mandatory) were acquired for nearly half of patients, but a relatively high assay failure rate limited the utility of these data. Germline analyses were performed on the subset of patients who had provided a blood sample with appropriate consent for this analysis. This added some additional homologous recombination repair mutation (HRRm) patients but tumor homologous recombination repair (HRR) status at this point was still unknown for 70% of patients. We therefore pursued analysis of baseline circulating tumor DNA (ctDNA). At the time of this work in early 2017, no commercial plasma assay covering all regions of all genes of interest was available. Analyses of ctDNA were initiated with an internal research use only (RUO) assay (AZ100) to meet timelines for database lock (DBL) (October 2017). To fill the remaining gaps in the HRR biomarker data, plasma samples were prioritized for analysis from patients with no tumor result and/or no HRR mutation identified via the germline assay. Results from these tumor, blood, and plasma analyses represent the "initial" dataset as previously reported by Clarke et al., 2018 [1] (described in the Results section in the main manuscript and Supplementary Figure S2). Subsequent to DBL, we completed analysis of available samples from patients with the AZ100 assay and also performed low-pass whole genome (LPWG) sequencing of the libraries from all samples with sufficient next-generation sequencing (NGS) library remaining. A subset of plasma samples from patients with tumor tissue results were subsequently analyzed by one or both external contract research organizations to support a separate assessment of those emerging assays.

Circulating Tumor DNA (ctDNA) Extraction/Purification

Blood samples for plasma ctDNA were collected at baseline (visit 2, pre-dose) into 8.5 mL K2 EDTA preparation tubes, mixed by 8–10 inversions and processed to plasma within 2 hours of collection. Processing involved centrifugation at 1300 × *g* for 10 minutes, aspiration of the plasma using a 5 mL syringe fitted with a needle, then filtration through a 0.8 µm syringe filter. Plasma was stored as approximately 1 mL aliquots at or below −70°C. For in-house analysis, extraction of ctDNA and subsequent analysis was performed in AstraZeneca Translational Medicine labs (Cambridge, UK). Up to 2 mL of plasma (range 0.7–2 mL) was used from each sample. Prior to extraction, plasma samples were spiked with a known quantity of a short synthetic non-human double-strand DNA sequence to enable later estimation of extraction efficiency via droplet digital polymerase chain reaction (PCR) [2]. Extraction was performed using a Maxwell RSC instrument and Maxwell RSC ccfDNA plasma kit (Promega Corporation, Southampton, UK). Maxwell RSC ccfDNA plasma kit quick protocol FB21212 was used with the exception that 55 µL Elution Buffer was used instead of the 60 µL specified in the protocol. Where available, 2 mL from each sample was processed as 2 × 1 mL per well and the two eluates combined prior to evaluation of extracted ctDNA. For samples with <1 mL or between 1 and 2 mL of plasma, the volume was made up to the nearest mL with elution buffer prior to extraction. Quantity and quality of the ctDNA obtained was assessed using both Qubit™ dsDNA HS Assay Kit (ThermoFisher Scientific, Waltham, MA, USA) and Agilent High Sensitivity ScreenTape (Agilent Technologies, Santa Clara, CA, USA) on the Agilent 4200 Tapestation. Purified ctDNA was transferred to DNA low-bind tubes (DNA LoBind®, Eppendorf or SC Micro Tube DNA LB, Sarstedt, Newton, NC, USA) and stored at 4°C overnight or at −20°C if not being used immediately. Extraction and purification of ctDNA

for externally run assays was performed by the selected vendors according to their own validated methods.

ctDNA Whole Genome Library Preparation

Whole genome libraries were prepared using the KAPA HyperPrep kit (Roche, Basel, Switzerland). Typically, all extracted ctDNA (3–650 ng [mean 37.5 ng, standard deviation 80 ng]) was subjected to library preparation, with an upper limit of 175 ng input per sample (see Results and Supplementary Table S3). The standard vendor's protocol (KR0161-v.5.16) was used with the following exceptions: (i) two reactions per sample; (ii) ligation was conducted for 2 hours instead of 15 minutes; and (iii) 0.7X AMPure beads (Beckman Coulter, High Wycombe, UK) volume (in relation to reaction volume) was used for post-ligation clean-up and 0.9X AMPure beads volume for post-amplification clean-up. Adapters sourced from Integrated DNA Technologies (IDT, Leuven, Belgium) with dual barcodes (eight nucleotides [nt] and six nt, respectively) were used at the concentrations recommended by the standard protocol. The adapters also contained a six-nt string of random bases (Unique Molecular Index, UMI) to allow for tagging of individual molecules. The number of PCR cycles used was dependent on the starting amount and varied between four and nine cycles. Libraries were quantified using D1000 ScreenTape on the Agilent 4200 Tapestation. In the absence of ctDNA, a viable plasma ctDNA library can still be obtained from the ubiquitous contaminating genomic DNA derived predominantly from leukocytes. Therefore, for the in-house AZ100 assay, plasma samples were considered "germline informative" if a viable sequencing library was generated and sequence data obtained to at least 100-fold mean depth. All but one library yielding AZ100 sequence data greatly exceeded this threshold (see Supplementary Table 4).

In-House Sequencing of ctDNA

Target enrichment utilized a custom xGen® Lockdown® probe panel (IDT) covering the full coding sequences of 112 genes (Supplementary Table S2), and with a sequencing footprint of ~0.66 Mb. Target enrichment was achieved following the IDT "Hybridization-capture of DNA libraries using xGen Lockdown probes and reagents" protocol (NGS-10021-PR, version 2) with the following exceptions: (i) libraries were pooled in multiples of six for hybridization (aiming for a total library input mass of 660 ng per hybridization); (ii) 2 µL of xGen Universal Blockers-TS Mix was user per reaction; (iii) after thermal denaturation, the DNA was snap chilled on ice for 1 minute prior to addition of probes; (iv) prior to hybridization, DNA and probes were incubated at 95°C for 2 minutes to ensure all molecules remained single stranded; (v) hybridization time was 20–23 hours; (vi) post-capture amplification utilized nine PCR cycles; and (vii) the final library was purified again with 0.8X AMPure beads volume (in relation to reaction volume). Prior to sequencing, the pools of libraries were quantified using KAPA Library Quantification kit for Illumina (Roche) on a ThermoFisher QuantStudio instrument (ThermoFisher Scientific). The final libraries were sequenced on a NextSeq 500 instrument (Illumina, Cambridge, UK), sequencing 12 samples per High Output v2 kit (300 cycles), typically yielding around 10 gigabases raw read data per sample. The panel and unique molecular indices enrichment methods were validated using commercial plasma samples harboring somatic alterations at known allele frequencies. LPWG sequencing, when performed, utilized the same NGS library created for the targeted sequencing but with sequencing performed on an Illumina HiSeq4000 instrument, sequencing nine samples per lane, yielding ~12 gigabases of raw data per sample.

Data Processing, Curation, and Visualization

In-house targeted ctDNA sequence data was analyzed using pipeline software bcbio-nextgen 1.0.4 [3]. Reads were aligned to the hg38 reference using bwa 0.7.15 [4], a quality control (QC) report was generated using multiqc [5], and sequencing duplicates for each UMI were collapsed into a single consensus read using Fgbio [6]. Variant calling was performed using VarDict [7] down to a variant allele frequency (VAF) of 0.1% (before filtering and curation) and variant effects annotated by snpEff [8]. Filtering of non-cancer variants (i.e., common polymorphisms) was performed as per VarDict best practice [7]. Copy number analysis for both exon- and whole-gene level was performed using Seq2C [9] for each gene in the panel. The change in the normalized Log2 values was used to determine potential copy number changes. Of the 112 genes covered by the in-house assay, variants in the 15 core HRR genes as well as a subset of genes expected to be commonly mutated in metastatic castrate-resistant prostate cancer (mCRPC) were prioritized for detailed review.

For small variants (single and multi-nucleotide variants and small indels), the following steps were employed to distinguish real events from false positives. Variants with a VAF <0.5% were excluded except for previously reported variants with likely pathogenic effects. The quality of each call was evaluated by visualization of mapped reads in the New Genome Browser (NGB) [10]. A variant was likely to be considered an artefact if one or more of the following features was observed: multiple additional nearby variants, Ns (undetermined basecalls) at or near the variant location at a similar or higher frequency to the variant, or many soft-clipped reads overlapping or close to the variant site. In addition, a variant was classified as a potential artefact if the position had low overall unique coverage (<100×) or strand bias (unequal representation of the variant in reads mapped to each strand) determined using an indicator from DFKZBias [11]. If a variant was a novel putative loss-of-function (LoF) mutation in a tumor suppressor and present in more than one subject, it was excluded. Variants with an allele frequency close to 50% or 100% were presumed likely germline (where matched germline data across the locus existed, we confirmed the presence of those calls). Variants present in at least one population group in ExAC [12] or GnomAD [13] at >0.5% were considered likely benign/benign in nature and excluded. Exploiting the use of deep sequencing and UMIs coupled with manual curation enabled us to call HRRm variants with confidence to as low as 0.6% VAF. For the purposes of assigning HRRm status, only putative variants considered likely to be deleterious to HRR gene function were retained. Variants were assessed for potential pathogenicity and relationship to disease or treatment response by reference to literature and public data sources and in line with American College of Medical Genetics and Genomics (ACMG) guidelines for classification of variants [14] and joint ACMG/American Society of Clinical Oncology (ASCO)/College of American Pathologists (CAP) guidelines for the interpretation and reporting of sequence variants in cancer [15].

For copy number alterations (CNAs), in an approach similar to that of Mayrhofer et al. [16], we supplemented our understanding of copy number events by also sequencing whole genome libraries at low depth (LPWG). We utilized ichorCNA [17] to estimate the percentage tumor content of the ctDNA libraries. Read count files were generated using readCounter from HMMCopy [18], using 1 Mb bins across all chromosomes, prior to running the ichorCNA R package. Log2 ratios from HMMCopy output were visualized within TIBCO Spotfire (TIBCO Software Inc., Palo Alto, CA, USA) and heatmaps were generated using the heatmap.2 [19] and dist [20] packages in R.

Color Genomics' germline targeted panel data was reviewed at the level of variant calls (pathogenic/likely pathogenic) in the nine HRR genes of interest covered by the assay.

When assessing mutations suspected as being potentially derived from clonal hematopoiesis (CH)-specific locations in the Binary Alignment Map (BAM) files were evaluated. Whole genome BAM files (hg19) from HLI Inc. (San Diego, CA, USA) were processed using bcbio-nextgen [3] on the DNANexus platform (DNANexus, Mountain View, CA, USA), with variants called by Genome Analysis Toolkit (GATK) [21]. Circular visualizations for figures were generated with BioCircos.R [22].

Criteria for Homologous Recombination Repair Wild Type (HRRwt) Classification

For patients without a tumor result but having a successful ctDNA result (targeted panel and/or LPWG) with no known deleterious/suspected deleterious mutation in the core 15 HRR genes, classification as HRR wild type (HRRwt) was based on the following: (i) detection of a variant in any assayed gene determined to be tumor derived (not germline or suspected related to CH) with high confidence at a VAF of ≥5% (approximately 10-fold higher than our internal panel assay sensitivity for small variants); (ii) detection of CNAs in targeted or LPWG data (assumption is that the tumor fraction must be >5% for such events to be detectable by our pipeline); or (iii) clear signal of an aberrant tumor genome in LPWG profile (evidence for various large-scale genomic gains/losses). For the last criterion, the assumption was that regardless of any specific copy number variant call, if the observed LPWG read profile indicated multiple deviations from the mean (diploid) signal, these must be derived from the patient's tumor and it is very likely the tumor fraction was ≥5%.

Tumor fraction was estimated based on the following criteria: (i) Highest VAF for any high confidence somatic mutation (in any gene in the AZ100 targeted assay) or (ii) IchorCNA estimate (only viable for samples with tumor fraction of 10% or above).

Clonal hematopoiesis and *CHEK2*

Clonal hematopoiesis (CH) describes the expansion of blood cells descended from a single hematopoietic stem cell. Steensma et al. [23] first described the term "Clonal Hematopoiesis of Indeterminate Potential" (CHIP) to describe the presence in the blood of expanded clones bearing somatic mutations in cancer driver genes detectable at over 2% VAF but where the individual showed no other signs of hematological malignancy. CH increases in prevalence with age and prior exposure to cytotoxic therapies [24]. As ctDNA assays become increasingly routine and sensitive, the potential for mis-assignment of variants as being of tumor origin, when in fact they may be derived from leakage of DNA from nucleated cells displaying CH, becomes a real concern. *CHEK2* is one gene implicated in CH, and in our ctDNA analysis we observed two different and apparently somatic variants in this gene, at around 11% and 1.7% VAF. Sequencing of a germline DNA sample (from peripheral blood mononuclear cells [PBMCs] collected at enrollment) from the former patient confirmed unequivocally that the variant was a product of CH. Germline sequencing of the latter patient's PBMC DNA sample was not deep enough to identify the low-frequency variant but we excluded it based on a suspicion it could also be related to CH.

Classification of Germline *CHEK2* Variants

We observed two cases of patients harboring the same deleterious *CHEK2* variant in their germline (*CHEK2* c.1100delC, p.T367fs*15). This variant is rare in the European (non-Finnish) population (~0.2% VAF) but somewhat more prevalent in Finnish individuals (~0.8% VAF), included in the Exome Aggregation Consortium (ExAC) [12], and various literature reports define it as a risk factor for familial breast cancer. Several recent references have noted a likely relationship for this variant with increased risk of prostate cancer [25] and of breast cancer in male Finnish patients [26], and a higher rate of the

variant has been reported in lethal prostate cancer cases of European American origin [27]. These variants have been included in our HRRm set.

The missense variant Ile157Thr was observed as a heterozygous variant in 7/102 (6.8%) tested patients with a germline sample available for testing. This variant is rare in EU (non-Finnish) subjects at 0.47% population VAF (ExAC), but is more prevalent in Finnish subjects (2.6% population VAF; ExAC). This variant has various reports relating it to cancer risk, including breast and prostate cancer [28-30]. The variant has been detected in families with Li-Fraumeni syndrome and is enriched in breast cancer patients from Finland [29]. This variant has multiple but conflicting interpretations in ClinVar (https://www.ncbi.nlm.nih.gov/clinvar/variation/5591/). Some laboratories classify this variant as pathogenic or likely pathogenic, but it has also been classified as a variant of uncertain significance (VUS) and a risk factor for cancer development but with lower penetrance than other *CHEK2* pathogenic variants. In assessing the balance of evidence for this variant, we conclude it should be classified as a VUS at this time and thus patients with I157T were not included in our HRRm set.

SUPPLEMENTAL RESULTS

Table S1. AZ100 gene list, hg38 gene coordinates and reference transcripts

Gene	Ensembl transcript ID	Chr location (GRCh38)
ABCB1	ENST00000265724.7	chr7:87504226-87600210
AKT1	ENST00000555528.5	chr14:104770320-104792680
AKT2	ENST00000392038.6	chr19:40233851-40265406
ALK	ENST00000389048.7	chr2:29193212-29920685
AR	ENST00000374690.7	chrX:67545099-67723883
ARAF	ENST00000290277.10	chrX:47562955-47571510
ATM	ENST00000278616.8	chr11:108227600-108365536
BARD1	ENST00000260947.8	chr2:214728662-214809614
BRAF	ENST00000288602.10	chr7:140719324-140924624
BRCA1	ENST00000357654.7	chr17:43045620-43124116
BRCA2	ENST00000380152.7	chr13:32316434-32398825
BRIP1	ENST00000259008.6	chr17:61683238-61861553
CARD11	ENST00000396946.8	chr7:2906620-2958562
CCND1	ENST00000227507.2	chr11:69641292-69651319
CCNE1	ENST00000262643.7	chr19:29812509-29823836
CD274	ENST00000381577.3	chr9:5456079-5467910
CDH1	ENST00000611625.4	chr16:68737381-68833514
CDK12	ENST00000447079.4	chr17:39462054-39531391
CDK4	ENST00000257904.10	chr12:57748510-57751738
CDK5	ENST00000485972.5	chr7:151053991-151057890

Gene	Ensembl transcript ID	Chr location (GRCh38)
CDK6	ENST00000265734.8	chr7:92615092-92833327
CDKN1B	ENST00000396340.1	chr12:12717837-12719005
CDKN2A	ENST00000304494.9	chr9:21968175-21994476
CDKN2B	ENST00000276925.6	chr9:22005937-22008993
CHEK1	ENST00000534070.5	chr11:125626740-125655332
CHEK2	ENST00000328354.10	chr22:28687881-28734741
CTLA4	ENST00000302823.7	chr2:203867936-203872819
EGFR	ENST00000275493.6	chr7:55019261-55205676
ERBB2	ENST00000269571.9	chr17:39699479-39728046
ERG	ENST00000398919.6	chr21:38379957-38575764
ESR1	ENST00000206249.7	chr6:151807898-152098969
ETV1	ENST00000405358.8	chr7:13895856-13989090
ETV4	ENST00000319349.9	chr17:43528511-43545651
ETV5	ENST00000306376.9	chr3:186048629-186108671
ETV6	ENST00000396373.8	chr12:11650083-11891053
EZH2	ENST00000320356.6	chr7:148807616-148850522
FANCL	ENST00000233741.8	chr2:58159722-58241325
FGFR1	ENST00000447712.6	chr8:38413596-38461160
FGFR2	ENST00000457416.6	chr10:121479561-121593823
FGFR3	ENST00000440486.6	chr4:1793928-1807309
FH	ENST00000366560.3	chr1:241497778-241519776
FOXA1	ENST00000250448.2	chr14:37591317-37594996
FRS2	ENST00000397997.6	chr12:69569003-69574960
GATA3	ENST00000379328.7	chr10:8055595-8074060
GNA11	ENST00000078429.8	chr19:3094624-3121204
GNAQ	ENST00000286548.8	chr9:77721297-78031287
GNAS	ENST00000371085.7	chr20:58840072-58910876
HGF	ENST00000222390.9	chr7:81702549-81769987
HRAS	ENST00000311189.7	chr11:532551-534326
INHBA	ENST00000242208.4	chr7:41689610-41700420
KEAP1	ENST00000171111.9	chr19:10486614-10500073
KEL	ENST00000355265.6	chr7:142941212-142962264
KIT	ENST00000288135.5	chr4:54657988-54738612
KRAS	ENST00000256078.8	chr12:25209471-25245454

Gene	Ensembl transcript ID	Chr location (GRCh38)
MAP2K1	ENST00000307102.9	chr15:66387327-66490618
MAP2K2	ENST00000262948.9	chr19:4090592-4123889
MAP2K4	ENST00000415385.7	chr17:12020884-12141263
MAP3K1	ENST00000399503.3	chr5:56815544-56893725
MAPK1	ENST00000215832.10	chr22:21769202-21867433
MAPK3	ENST00000263025.8	chr16:30116608-30123244
MCL1	ENST00000369026.2	chr1:150577307-150579546
MDM2	ENST00000258149.9	chr12:68808424-68839861
MET	ENST00000397752.7	chr7:116699017-116796125
MLH1	ENST00000231790.6	chr3:36993545-37050689
MSH2	ENST00000233146.6	chr2:47403177-47512488
MSH6	ENST00000234420.9	chr2:47783183-47806879
MTOR	ENST00000361445.8	chr1:11106572-11259451
MYC	ENST00000613283.1	chr8:127736548-127740977
NF1	ENST00000358273.8	chr17:31095279-31374203
NF2	ENST00000338641.8	chr22:29603995-29694837
NFE2L2	ENST00000397062.7	chr2:177230739-177264614
NRAS	ENST00000369535.4	chr1:114708474-114716164
NUDT1	ENST00000343985.8	chr7:2244524-2251034
PALB2	ENST00000261584.8	chr16:23603443-23641193
PARP1	ENST00000366794.9	chr1:226361440-226407989
PBRM1	ENST00000296302.11	chr3:52548029-52681782
PDCD1	ENST00000334409.9	chr2:241850997-241858860
PDGFRA	ENST00000257290.9	chr4:54258740-54295318
PIK3CA	ENST00000263967.3	chr3:179198821-179234408
PIK3CB	ENST00000289153.6	chr3:138655337-138759377
PIK3CG	ENST00000359195.3	chr7:106867533-106905428
PIK3R1	ENST00000521381.5	chr5:68226662-68297626
PIN1	ENST00000247970.8	chr19:9835314-9849204
PMS2	ENST00000265849.11	chr7:5973350-6009067
PPP2R2A	ENST00000380737.7	chr8:26291763-26370453
PTEN	ENST00000371953.7	chr10:87864448-87965499
RAC1	ENST00000356142.4	chr7:6374700-6402501
RAD51B	ENST00000487861.5	chr14:67823525-68683000

Gene	Ensembl transcript ID	Chr location (GRCh38)
RAD51C	ENST00000337432.8	chr17:58692595-58734229
RAD51D	ENST00000345365.10	chr17:35100934-35119632
RAD54L	ENST00000371975.8	chr1:46248347-46278297
RAF1	ENST00000442415.6	chr3:12584465-12618737
RASA1	ENST00000274376.10	chr5:87268385-87390901
RB1	ENST00000267163.4	chr13:48303842-48480094
RET	ENST00000355710.7	chr10:43077177-43128310
RHEB	ENST00000262187.9	chr7:151467105-151519545
RICTOR	ENST00000296782.9	chr5:38942280-39074412
RIT1	ENST00000368322.7	chr1:155900383-155910941
ROS1	ENST00000368508.7	chr6:117288466-117425714
RUNDC3B	ENST00000338056.7	chr7:87628764-87830077
SETD2	ENST00000409792.3	chr3:47017053-47164034
SLC25A40	ENST00000341119.9	chr7:87836245-87858749
SMO	ENST00000249373.7	chr7:129189057-129212477
SPOP	ENST00000347630.6	chr17:49600330-49622831
STK11	ENST00000326873.11	chr19:1206878-1228137
TMPRSS2	ENST00000398585.7	chr21:41466087-41508036
TP53	ENST00000269305.8	chr17:7669589-7676608
TRRAP	ENST00000359863.8	chr7:98881140-99012392
TSC1	ENST00000298552.7	chr9:132896194-132928976
TSC2	ENST00000219476.7	chr16:2047763-2088647
XRCC2	ENST00000359321.1	chr7:152648582-152676119
ZBTB16	ENST00000335953.8	chr11:114063274-114250560

Table S2. Pass and fail results for all samples across all assays performed at (A) initial analysis and (B) final analysis

	Tumor	Germline		Plasma (ctDNA)											HRRm		
Patient no.	Tumor (FoundationOne)	Germline (Color Genomics)	Germline (HLI whole genome)	ctDNA (AZ100)	ctDNA LPWG	GH OMNI ctDNA result	RB ctDNA result	FMI ACT v2	ANY ctDNA Result	Evaluable ctDNA (high confidence somatic variant(s) in ctDNA NGS outputs)	ctDNA fraction ≤1%	ctDNA fraction between 1 and 5%	ctDNA fraction ≥5%	Data from at least one source	Deleterious in BRCA1/BRCA2/ATM (any source)	Deleterious in any of 15 HRR genes (any source)	Overall HRRm status
**A. Initial analysis**																	
1	F	Y		N					N	N	N	N	N	Y	Y	Y	BRCA/ATMm
2	Y	N		N					N	N	N	N	N	Y			wt in tissue
3	Y	Y		N					N	N	N	N	N	Y			wt in tissue
4	N	N		Y					Y	Y	N	N	Y	Y			wt in plasma
5	Y	Y		N					N	N	N	N	N	Y		Y	Other HRRm (tail)
6	N	Y		F					F	N	N	N	N	Y			Partially characterized
7	F	N		Y					Y	Y	N	N	Y	Y			wt in plasma
8	N	N		Y					Y	Y	N	N	Y	Y			wt in plasma
9	N	Y		Y					Y	N	N	N	N	Y			Partially characterized
10	F	Y		Y					Y	Y	N	N	Y	Y		Y	Other HRRm (tail)
11	Y	Y		N					N	N	N	N	N	Y			wt in tissue
12	Y	Y		N					N	N	N	N	N	Y			wt in tissue

	Tumor	Germline		Plasma (ctDNA)											HRRm		
Patient no.	Tumor (FoundationOne)	Germline (Color Genomics)	Germline (HLI whole genome)	ctDNA (AZ100)	ctDNA LPWG	GH OMNI ctDNA result	RB ctDNA result	FMI ACT v2	ANY ctDNA Result	Evaluable ctDNA (high confidence somatic variant(s) in ctDNA NGS outputs)	ctDNA fraction ≤1%	ctDNA fraction between 1 and 5%	ctDNA fraction ≥5%	Data from at least one source	Deleterious in BRCA1/BRCA2/ATM (any source)	Deleterious in any of 15 HRR genes (any source)	Overall HRRm status
13	Y	Y		N					N	N	N	N	N	Y			wt in tissue
14	N	Y		Y					Y	N	N	N	N	Y			Partially characterized
15	Y	N		N					N	N	N	N	N	Y			wt in tissue
16	N	N		Y					Y	N	N	N	N	Y			Partially characterized
17	Y	Y		N					N	N	N	N	N	Y			wt in tissue
18	Y	Y		N					N	N	N	N	N	Y			wt in tissue
19	N	N		Y					Y	Y	N	Y	N	Y			Partially characterized
20	Y	N		N					N	N	N	N	N	Y			wt in tissue
21	Y	N		N					N	N	N	N	N	Y			wt in tissue
22	N	Y		Y					Y	Y	N	Y	N	Y			Partially characterized
23	F	Y		Y					Y	N	N	N	N	Y			Partially characterized
24	Y	N		N					N	N	N	N	N	Y			wt in tissue
25	Y	Y		N					N	N	N	N	N	Y			wt in tissue
26	N	Y		Y					Y	Y	N	N	Y	Y			wt in plasma

	Tumor	Germline		Plasma (ctDNA)											HRRm		
Patient no.	Tumor (FoundationOne)	Germline (Color Genomics)	Germline (HLI whole genome)	ctDNA (AZ100)	ctDNA LPWG	GH OMNI ctDNA result	RB ctDNA result	FMI ACT v2	ANY ctDNA Result	Evaluable ctDNA (high confidence somatic variant(s) in ctDNA NGS outputs)	ctDNA fraction ≤1%	ctDNA fraction between 1 and 5%	ctDNA fraction ≥5%	Data from at least one source	Deleterious in BRCA1/BRCA2/ATM (any source)	Deleterious in any of 15 HRR genes (any source)	Overall HRRm status
27	F	N		Y					Y	Y	N	N	Y	Y	Y	Y	BRCA/ATMm
28	N	Y		Y					Y	Y	N	Y	N	Y			Partially characterized
29	F	Y		Y					Y	Y	N	N	Y	Y			wt in plasma
30	F	Y		Y					Y	Y	N	N	Y	Y			wt in plasma
31	Y	Y		N					N	N	N	N	N	Y			wt in tissue
32	Y	Y		N					N	N	N	N	N	Y			wt in tissue
33	Y	Y		N					N	N	N	N	N	Y			wt in tissue
34	F	Y		Y					Y	Y	N	N	Y	Y			wt in plasma
35	Y	Y		N					N	N	N	N	N	Y			wt in tissue
36	N	Y		Y					Y	N	N	N	N	Y			Partially characterized
37	N	Y		Y					Y	N	N	N	N	Y			Partially characterized
38	N	N		F					F	N	N	N	N	N			Partially characterized
39	F	N		Y					Y	N	N	N	N	Y			Partially characterized
40	F	Y		Y					Y	N	N	N	N	Y			Partially characterized

	Tumor	Germline		Plasma (ctDNA)											HRRm		
Patient no.	Tumor (FoundationOne)	Germline (Color Genomics)	Germline (HLI whole genome)	ctDNA (AZ100)	ctDNA LPWG	GH OMNI ctDNA result	RB ctDNA result	FMI ACT v2	ANY ctDNA Result	Evaluable ctDNA (high confidence somatic variant(s) in ctDNA NGS outputs)	ctDNA fraction ≤1%	ctDNA fraction between 1 and 5%	ctDNA fraction ≥5%	Data from at least one source	Deleterious in BRCA1/BRCA2/ATM (any source)	Deleterious in any of 15 HRR genes (any source)	Overall HRRm status
41	N	N		N					N	N	N	N	N	N			Partially characterized
42	N	N		Y					Y	Y	N	N	Y	Y			wt in plasma
43	Y	N		N					N	N	N	N	N	Y			wt in tissue
44	F	N		N					N	N	N	N	N	N			Partially characterized
45	N	N		Y					Y	Y	N	N	Y	Y			wt in plasma
46	Y	N		N					N	N	N	N	N	Y	Y	Y	BRCA/ATMm
47	N	N		Y					Y	N	N	N	N	Y			Partially characterized
48	F	Y		Y					Y	N	N	N	N	Y			Partially characterized
49	F	Y		Y					Y	N	N	N	N	Y			Partially characterized
50	Y	Y		N					N	N	N	N	N	Y			wt in tissue
51	Y	Y		N					N	N	N	N	N	Y			wt in tissue
52	N	Y		Y					Y	N	N	N	N	Y			Partially characterized
53	Y	N		Y					Y	Y	N	N	Y	Y			wt in tissue
54	Y	N		N					N	N	N	N	N	Y			wt in tissue

	Tumor	Germline		Plasma (ctDNA)											HRRm		
Patient no.	Tumor (FoundationOne)	Germline (Color Genomics)	Germline (HLI whole genome)	ctDNA (AZ100)	ctDNA LPWG	GH OMNI ctDNA result	RB ctDNA result	FMI ACT v2	ANY ctDNA Result	Evaluable ctDNA (high confidence somatic variant(s) in ctDNA NGS outputs)	ctDNA fraction ≤1%	ctDNA fraction between 1 and 5%	ctDNA fraction ≥5%	Data from at least one source	Deleterious in BRCA1/BRCA/ATM (any source)	Deleterious in any of 15 HRR genes (any source)	Overall HRRm status
55	F	N		Y					Y	Y	N	N	Y	Y			wt in plasma
56	F	N		Y					Y	Y	N	N	Y	Y	Y	Y	BRCA/ATMm
57	F	Y		F					F	N	N	N	N	Y			Partially characterized
58	F	Y		F					F	N	N	N	N	Y			Partially characterized
59	N	Y		Y					Y	Y	N	N	Y	Y			wt in plasma
60	N	N		Y					Y	N	N	N	N	Y			Partially characterized
61	N	N		Y					Y	N	N	N	N	Y			Partially characterized
62	Y	Y		Y					Y	Y	N	N	Y	Y			wt in tissue
63	F	Y		Y					Y	Y	N	N	Y	Y			wt in plasma
64	Y	Y		N					N	N	N	N	N	Y			wt in tissue
65	N	Y		N					N	N	N	N	N	Y		Y	Other HRRm (tail)
66	Y	Y		N					N	N	N	N	N	Y	Y	Y	BRCA/ATMm
67	N	Y		Y					Y	N	N	N	N	Y			Partially characterized
68	N	Y		Y					Y	Y	N	Y	N	Y			Partially characterized

	Tumor	Germline		Plasma (ctDNA)											HRRm		
Patient no.	Tumor (FoundationOne)	Germline (Color Genomics)	Germline (HLI whole genome)	ctDNA (AZ100)	ctDNA LPWG	GH OMNI ctDNA result	RB ctDNA result	FMI ACT v2	ANY ctDNA Result	Evaluable ctDNA (high confidence somatic variant(s) in ctDNA NGS outputs)	ctDNA fraction ≤1%	ctDNA fraction between 1 and 5%	ctDNA fraction ≥5%	Data from at least one source	Deleterious in BRCA1/BRCA/ATM (any source)	Deleterious in any of 15 HRR genes (any source)	Overall HRRm status
69	N	Y		Y					Y	Y	N	N	Y	Y			wt in plasma
70	N	Y		Y					Y	Y	N	N	Y	Y			wt in plasma
71	N	Y		Y					Y	Y	N	N	Y	Y	Y	Y	BRCA/ATMm
72	N	Y		Y					Y	Y	N	N	Y	Y			wt in plasma
73	N	Y		Y					Y	Y	N	Y	N	Y			Partially characterized
74	N	Y		Y					Y	Y	N	N	Y	Y			wt in plasma
75	Y	Y		N					N	N	N	N	N	Y			wt in tissue
76	N	Y		Y					Y	N	N	N	N	Y			Partially characterized
77	N	Y		Y					Y	Y	N	N	Y	Y			wt in plasma
78	N	Y		Y					Y	N	N	N	N	Y			Partially characterized
79	N	Y		Y					Y	Y	N	N	Y	Y			wt in plasma
80	N	Y		Y					Y	Y	N	N	Y	Y			wt in plasma
81	F	N		Y					Y	Y	N	Y	N	Y			Partially characterized
82	N	N		N					N	N	N	N	N	N			Partially characterized

	Tumor	Germline		Plasma (ctDNA)											HRRm		
Patient no.	Tumor (FoundationOne)	Germline (Color Genomics)	Germline (HLI whole genome)	ctDNA (AZ100)	ctDNA LPWG	GH OMNI ctDNA result	RB ctDNA result	FMI ACT v2	ANY ctDNA Result	Evaluable ctDNA (high confidence somatic variant(s) in ctDNA NGS outputs)	ctDNA fraction ≤1%	ctDNA fraction between 1 and 5%	ctDNA fraction ≥5%	Data from at least one source	Deleterious in BRCA1/BRCA2/ATM (any source)	Deleterious in any of 15 HRR genes (any source)	Overall HRRm status
83	Y	N		N					N	N	N	N	N	Y			wt in tissue
84	N	Y		N					N	N	N	N	N	Y			Partially characterized
85	Y	N		N					N	N	N	N	N	Y			wt in tissue
86	N	Y		Y					Y	Y	N	N	Y	Y		Y	Other HRRm (tail)
87	N	Y		Y					Y	Y	N	N	Y	Y		Y	Other HRRm (tail)
88	N	Y		Y					Y	N	N	N	N	Y			Partially characterized
89	N	Y		Y					Y	Y	N	N	Y	Y			wt in plasma
90	N	Y		Y					Y	Y	N	N	Y	Y			wt in plasma
91	N	Y		N					N	N	N	N	N	Y	Y	Y	BRCA/ATMm
92	N	Y		N					N	N	N	N	N	Y		Y	Other HRRm (tail)
93	N	Y		Y					Y	Y	N	Y	N	Y			Partially characterized
94	N	Y		Y					Y	N	N	N	N	Y			Partially characterized
95	F	Y		Y					Y	Y	N	N	Y	Y			wt in plasma
96	N	N		Y					Y	Y	N	N	Y	Y			wt in plasma

	Tumor	Germline		Plasma (ctDNA)											HRRm		
Patient no.	Tumor (FoundationOne)	Germline (Color Genomics)	Germline (HLI whole genome)	ctDNA (AZ100)	ctDNA LPWG	GH OMNI ctDNA result	RB ctDNA result	FMI ACT v2	ANY ctDNA Result	Evaluable ctDNA (high confidence somatic variant(s) in ctDNA NGS outputs)	ctDNA fraction ≤1%	ctDNA fraction between 1 and 5%	ctDNA fraction ≥5%	Data from at least one source	Deleterious in BRCA1/BRCA2/ATM (any source)	Deleterious in any of 15 HRR genes (any source)	Overall HRRm status
97	N	N		Y					Y	Y	N	N	Y	Y			wt in plasma
98	N	Y		Y					Y	Y	N	Y	N	Y			Partially characterized
99	N	Y		Y					Y	Y	N	N	Y	Y			Partially characterized
100	N	Y		Y					Y	Y	N	N	Y	Y	Y	Y	BRCA/ATMm
101	N	Y		Y					Y	Y	Y	N	N	Y	Y	Y	BRCA/ATMm
102	N	Y		Y					Y	Y	N	N	Y	Y			wt in plasma
103	N	Y		Y					Y	Y	N	N	Y	Y	Y	Y	BRCA/ATMm
104	N	Y		Y					Y	N	N	N	N	Y			Partially characterized
105	N	Y		Y					Y	Y	N	N	Y	Y	Y	Y	BRCA/ATMm
106	N	Y		Y					Y	Y	N	N	Y	Y			wt in plasma
107	N	Y		Y					Y	Y	N	Y	N	Y			Partially characterized
108	N	Y		Y					Y	Y	N	N	Y	Y			wt in plasma
109	N	Y		F					F	N	N	N	N	Y			Partially characterized
110	N	Y		Y					Y	N	N	N	N	Y			Partially characterized

	Tumor	Germline		Plasma (ctDNA)											HRRm		
Patient no.	Tumor (FoundationOne)	Germline (Color Genomics)	Germline (HLI whole genome)	ctDNA (AZ100)	ctDNA LPWG	GH OMNI ctDNA result	RB ctDNA result	FMI ACT v2	ANY ctDNA Result	Evaluable ctDNA (high confidence somatic variant(s) in ctDNA NGS outputs)	ctDNA fraction ≤1%	ctDNA fraction between 1 and 5%	ctDNA fraction ≥5%	Data from at least one source	Deleterious in BRCA1/BRCA2/ATM (any source)	Deleterious in any of 15 HRR genes (any source)	Overall HRRm status
111	N	Y		Y					Y	Y	Y	N	N	Y			Partially characterized
112	N	Y		Y					Y	Y	N	N	Y	Y			wt in plasma
113	N	N		Y					Y	Y	N	N	Y	Y		Y	Other HRRm (tail)
114	N	N		Y					Y	Y	N	N	Y	Y		Y	Other HRRm (tail)
115	Y	N		N					N	N	N	N	N	Y			wt in tissue
116	Y	N		N					N	N	N	N	N	Y			wt in tissue
117	N	Y		Y					Y	N	N	N	N	Y			Partially characterized
118	N	Y		Y					Y	Y	N	N	Y	Y			wt in plasma
119	N	N		Y					Y	Y	Y	N	N	Y			Partially characterized
120	F	Y		Y					Y	N	N	N	N	Y			Partially characterized
121	N	N		N					N	N	N	N	N	N			Partially characterized
122	N	Y		Y					Y	Y	N	N	Y	Y			wt in plasma
123	N	Y		Y					Y	Y	N	N	N	Y			Partially characterized
124	N	N		N					N	N	N	N	N	N			Partially characterized

	Tumor	Germline		Plasma (ctDNA)											HRRm		
Patient no.	Tumor (FoundationOne)	Germline (Color Genomics)	Germline (HLI whole genome)	ctDNA (AZ100)	ctDNA LPWG	GH OMNI ctDNA result	RB ctDNA result	FMI ACT v2	ANY ctDNA Result	Evaluable ctDNA (high confidence somatic variant(s) in ctDNA NGS outputs)	ctDNA fraction ≤1%	ctDNA fraction between 1 and 5%	ctDNA fraction ≥5%	Data from at least one source	Deleterious in BRCA1/BRCA2/ATM (any source)	Deleterious in any of 15 HRR genes (any source)	Overall HRRm status
125	Y	Y		N					N	N	N	N	N	Y			wt in tissue
126	F	Y		Y					Y	Y	N	N	Y	Y			wt in plasma
127	F	Y		Y					Y	Y	N	N	Y	Y			wt in plasma
128	N	Y		Y					Y	Y	Y	N	N	Y			Partially characterized
129	Y	Y		N					N	N	N	N	N	Y			wt in tissue
130	F	Y		Y					Y	Y	N	N	Y	Y			wt in plasma
131	Y	Y		N					N	N	N	N	N	Y			wt in tissue
132	Y	Y		Y					Y	Y	Y	N	N	Y			wt in tissue
133	F	Y		Y					Y	Y	N	N	Y	Y			wt in plasma
134	Y	Y		N					N	N	N	N	N	Y			wt in tissue
135	F	Y		Y					Y	Y	N	Y	N	Y			Partially characterized
136	F	Y		Y					Y	Y	N	Y	N	Y	Y	Y	BRCA/ATMm
137	F	Y		Y					Y	N	N	N	N	Y			Partially characterized
138	F	N		Y					Y	Y	N	N	N	Y			Partially characterized

	**Tumor**	**Germline**		**Plasma (ctDNA)**											**HRRm**		
Patient no.	Tumor (FoundationOne)	Germline (Color Genomics)	Germline (HLI whole genome)	ctDNA (AZ100)	ctDNA LPWG	GH OMNI ctDNA result	RB ctDNA result	FMI ACT v2	ANY ctDNA Result	Evaluable ctDNA (high confidence somatic variant(s) in ctDNA NGS outputs)	ctDNA fraction ≤1%	ctDNA fraction between 1 and 5%	ctDNA fraction ≥5%	Data from at least one source	Deleterious in BRCA1/BRCA/ATM (any source)	Deleterious in any of 15 HRR genes (any source)	Overall HRRm status
139	F	Y		Y					Y	Y	N	Y	N	Y			Partially characterized
140	Y	Y		N					N	N	N	N	N	Y			wt in tissue
141	N	Y		N					N	N	N	N	N	Y	Y	Y	BRCA/ATMm
142	Y	Y		N					N	N	N	N	N	Y			wt in tissue
YES	**38**	**102**	**0**	**91**	**0**	**0**	**0**	**0**	**91**	**67**	**5**	**12**	**48**	**136**	**13**	**21**	**13** BRCA/ATMm
NO	**74**	**40**	**0**	**46**	**0**	**0**	**0**	**0**	**46**	**75**				**6**			**8** other HRRm (tail)
FAIL	**30**	**0**	**0**	**5**	**0**	**0**	**0**	**0**	**5**	**0**				**0**			**35** wt in tissue

86 total wt in plasma and unknown = "partially characterized" for interim set

B. Final analysis

1	F	Y	Y	F	N	N	N	N	N	N	N	N	N	Y	Y	Y	BRCA/ATMm
2	Y	N	N	N	N	Y	Y	N	Y	Y	N	N	Y	Y			wt in tissue
3	Y	Y	Y	N	N	Y	Y	N	Y	Y	N	Y	N	Y			wt in tissue
4	N	N	N	Y	N	N	N	N	Y	Y	N	N	Y	Y			wt in plasma
5	Y	Y	Y	N	N	Y	Y	N	Y	Y	N	N	Y	Y		Y	Other HRRm (tail)

	Tumor	Germline		Plasma (ctDNA)											HRRm		
Patient no.	Tumor (FoundationOne)	Germline (Color Genomics)	Germline (HLI whole genome)	ctDNA (AZ100)	ctDNA LPWG	GH OMNI ctDNA result	RB ctDNA result	FMI ACT v2	ANY ctDNA Result	Evaluable ctDNA (high confidence somatic variant(s) in ctDNA NGS outputs)	ctDNA fraction ≤1%	ctDNA fraction between 1 and 5%	ctDNA fraction ≥5%	Data from at least one source	Deleterious in BRCA1/BRCA2/ATM (any source)	Deleterious in any of 15 HRR genes (any source)	Overall HRRm status
6	N	Y	Y	F	N	N	N	N	N	N	N	N	N	Y			Partially characterized
7	F	N	N	Y	Y	N	N	N	Y	Y	N	N	Y	Y			wt in plasma
8	N	N	N	Y	N	N	N	N	Y	Y	N	N	Y	Y			wt in plasma
9	N	Y	Y	Y	Y	N	N	N	Y	N	N	N	N	Y			Partially characterized
10	F	Y	Y	Y	Y	N	N	N	Y	Y	N	N	Y	Y		Y	Other HRRm (tail)
11	Y	Y	Y	N	N	Y	Y	N	Y	Y	N	N	Y	Y			wt in tissue
12	Y	Y	Y	Y	Y	N	N	N	Y	Y	N	N	Y	Y			wt in tissue
13	Y	Y	Y	N	N	Y	Y	N	Y	Y	N	N	Y	Y			wt in tissue
14	N	Y	Y	Y	Y	N	N	N	Y	N	N	N	N	Y			Partially characterized
15	Y	N	N	N	N	Y	Y	N	Y	Y	N	N	Y	Y			wt in tissue
16	N	N	N	Y	Y	N	N	N	Y	N	N	N	N	Y			Partially characterized
17	Y	Y	Y	N	N	F	F	N	N	N	N	N	N	Y			wt in tissue
18	Y	Y	Y	N	N	F	Y	N	Y	N	N	N	N	Y			wt in tissue
19	N	N	N	Y	Y	N	N	N	Y	Y	N	N	Y	Y			wt in plasma

	Tumor	Germline		Plasma (ctDNA)											HRRm		
Patient no.	Tumor (FoundationOne)	Germline (Color Genomics)	Germline (HLI whole genome)	ctDNA (AZ100)	ctDNA LPWG	GH OMNI ctDNA result	RB ctDNA result	FMI ACT v2	ANY ctDNA Result	Evaluable ctDNA (high confidence somatic variant(s) in ctDNA NGS outputs)	ctDNA fraction ≤1%	ctDNA fraction between 1 and 5%	ctDNA fraction ≥5%	Data from at least one source	Deleterious in BRCA1/BRCA1/ATM (any source)	Deleterious in any of 15 HRR genes (any source)	Overall HRRm status
20	Y	N	N	N	N	Y	F	N	Y	Y	N	Y	N	Y			wt in tissue
21	Y	N	N	N	N	Y	Y	N	Y	Y	N	Y	N	Y			wt in tissue
22	N	Y	Y	Y	Y	N	N	N	Y	Y	N	Y	N	Y			Partially characterized
23	F	Y	Y	Y	Y	N	N	N	Y	N	N	N	N	Y			Partially characterized
24	Y	N	N	N	N	Y	Y	N	Y	Y	N	Y	N	Y			wt in tissue
25	Y	Y	Y	N	N	Y	F	N	Y	Y	N	N	Y	Y			wt in tissue
26	N	Y	Y	Y	Y	N	N	N	Y	Y	N	N	Y	Y			wt in plasma
27	F	N	N	Y	Y	N	N	Y	Y	Y	N	N	Y	Y	Y	Y	BRCA/ATMm
28	N	Y	Y	Y	Y	N	N	N	Y	Y	N	Y	N	Y			Partially characterized
29	F	Y	Y	Y	Y	N	N	N	Y	Y	N	N	Y	Y			wt in plasma
30	F	Y	Y	Y	Y	N	N	N	Y	Y	N	N	Y	Y			wt in plasma
31	Y	Y	Y	N	N	Y	Y	N	Y	Y	N	N	Y	Y			wt in tissue
32	Y	Y	Y	N	N	Y	Y	N	Y	Y	Y	N	N	Y			wt in tissue
33	Y	Y	Y	Y	Y	N	N	N	Y	Y	N	N	Y	Y		Y	Other HRRm (tail)

	Tumor	Germline		Plasma (ctDNA)											HRRm		
Patient no.	Tumor (FoundationOne)	Germline (Color Genomics)	Germline (HLI whole genome)	ctDNA (AZ100)	ctDNA LPWG	GH OMNI ctDNA result	RB ctDNA result	FMI ACT v2	ANY ctDNA Result	Evaluable ctDNA (high confidence somatic variant(s) in ctDNA NGS outputs)	ctDNA fraction ≤1%	ctDNA fraction between 1 and 5%	ctDNA fraction ≥5%	Data from at least one source	Deleterious in BRCA1/BRCA/ATM (any source)	Deleterious in any of 15 HRR genes (any source)	Overall HRRm status
34	F	Y	Y	Y	Y	N	N	N	Y	Y	N	N	Y	Y			wt in plasma
35	Y	Y	Y	Y	Y	N	N	N	Y	Y	N	N	Y	Y			wt in tissue
36	N	Y	Y	Y	Y	N	N	N	Y	N	N	N	N	Y			Partially characterized
37	N	Y	Y	Y	Y	N	N	N	Y	N	N	N	N	Y			Partially characterized
38	N	N	N	F	N	N	N	N	N	N	N	N	N	N			Partially characterized
39	F	N	N	Y	Y	N	N	N	Y	N	N	N	N	Y			Partially characterized
40	F	Y	Y	Y	Y	N	N	N	Y	N	N	N	N	Y			Partially characterized
41	N	N	N	N	N	N	N	N	N	N	N	N	N	N			Partially characterized
42	N	N	N	Y	Y	N	N	N	Y	Y	N	N	Y	Y			wt in plasma
43	Y	N	N	Y	N	N	N	N	Y	N	N	N	N	Y			wt in tissue
44	F	N	N	N	N	N	N	N	N	N	N	N	N	N			Partially characterized
45	N	N	N	Y	Y	N	N	N	Y	Y	N	N	Y	Y			wt in plasma
46	Y	N	N	Y	Y	N	N	N	Y	N	N	N	N	Y	Y	Y	BRCA/ATMm
47	N	N	N	Y	Y	N	N	N	Y	N	N	N	N	Y			Partially characterized

	Tumor	Germline		Plasma (ctDNA)											HRRm		
Patient no.	Tumor (FoundationOne)	Germline (Color Genomics)	Germline (HLI whole genome)	ctDNA (AZ100)	ctDNA LPWG	GH OMNI ctDNA result	RB ctDNA result	FMI ACT v2	ANY ctDNA Result	Evaluable ctDNA (high confidence somatic variant(s) in ctDNA NGS outputs)	ctDNA fraction ≤1%	ctDNA fraction between 1 and 5%	ctDNA fraction ≥5%	Data from at least one source	Deleterious in BRCA1/BRCA2/ATM (any source)	Deleterious in any of 15 HRR genes (any source)	Overall HRRm status
48	F	Y	Y	Y	Y	N	N	N	Y	N	N	N	N	Y			Partially characterized
49	F	Y	Y	Y	Y	N	N	N	Y	N	N	N	N	Y			Partially characterized
50	Y	Y	Y	N	N	Y	F	N	Y	Y	Y	N	N	Y			wt in tissue
51	Y	Y	Y	Y	Y	N	N	N	Y	Y	N	N	Y	Y			wt in tissue
52	N	Y	Y	Y	Y	N	N	N	Y	N	N	N	N	Y			Partially characterized
53	Y	N	N	Y	Y	N	N	N	Y	Y	N	N	Y	Y			wt in tissue
54	Y	N	N	Y	Y	N	N	N	Y	N	N	N	N	Y			wt in tissue
55	F	N	N	Y	N	N	N	Y	Y	Y	N	N	Y	Y			wt in plasma
56	F	N	N	Y	Y	N	N	Y	Y	Y	N	N	Y	Y	Y	Y	BRCA/ATMm
57	F	Y	Y	F	N	N	N	N	N	N	N	N	N	Y			Partially characterized
58	F	Y	Y	F	N	N	N	N	N	N	N	N	N	Y			Partially characterized
59	N	Y	Y	Y	Y	N	N	N	Y	Y	N	N	Y	Y			wt in plasma
60	N	N	N	Y	Y	N	N	N	Y	N	N	N	N	Y			Partially characterized
61	N	N	N	Y	Y	N	N	N	Y	Y	N	N	Y	Y			wt in plasma

	Tumor	Germline		Plasma (ctDNA)											HRRm		
Patient no.	Tumor (FoundationOne)	Germline (Color Genomics)	Germline (HLI whole genome)	ctDNA (AZ100)	ctDNA LPWG	GH OMNI ctDNA result	RB ctDNA result	FMI ACT v2	ANY ctDNA Result	Evaluable ctDNA (high confidence somatic variant(s) in ctDNA NGS outputs)	ctDNA fraction ≤1%	ctDNA fraction between 1 and 5%	ctDNA fraction ≥5%	Data from at least one source	Deleterious in BRCA1/BRCA/ATM (any source)	Deleterious in any of 15 HRR genes (any source)	Overall HRRm status
62	Y	Y	Y	Y	Y	N	N	N	Y	Y	N	N	Y	Y			wt in tissue
63	F	Y	Y	Y	Y	N	N	N	Y	Y	N	N	Y	Y			wt in plasma
64	Y	Y	Y	Y	Y	N	N	N	Y	Y	Y	N	N	Y			wt in tissue
65	N	Y	Y	Y	Y	N	Y	N	Y	Y	N	Y	N	Y		Y	Other HRRm (tail)
66	Y	Y	Y	Y	Y	N	N	N	Y	Y	N	N	Y	Y	Y	Y	BRCA/ATMm
67	N	Y	Y	Y	Y	N	N	N	Y	N	N	N	N	Y			Partially characterized
68	N	Y	Y	Y	Y	N	N	N	Y	Y	N	Y	N	Y			Partially characterized
69	N	Y	Y	Y	Y	N	N	N	Y	Y	N	N	Y	Y			wt in plasma
70	N	Y	Y	Y	Y	N	N	N	Y	Y	N	N	Y	Y			wt in plasma
71	N	Y	Y	Y	Y	N	N	N	Y	Y	N	N	Y	Y	Y	Y	BRCA/ATMm
72	N	Y	Y	Y	N	N	N	N	Y	Y	N	N	Y	Y			wt in plasma
73	N	Y	Y	Y	Y	N	N	N	Y	Y	N	Y	N	Y			Partially characterized
74	N	Y	Y	Y	Y	N	N	N	Y	Y	N	N	Y	Y			wt in plasma
75	Y	Y	Y	Y	Y	N	N	N	Y	N	N	N	N	Y			wt in tissue

	Tumor	Germline		Plasma (ctDNA)											HRRm		
Patient no.	Tumor (FoundationOne)	Germline (Color Genomics)	Germline (HLI whole genome)	ctDNA (AZ100)	ctDNA LPWG	GH OMNI ctDNA result	RB ctDNA result	FMI ACT v2	ANY ctDNA Result	Evaluable ctDNA (high confidence somatic variant(s) in ctDNA NGS outputs)	ctDNA fraction ≤1%	ctDNA fraction between 1 and 5%	ctDNA fraction ≥5%	Data from at least one source	Deleterious in BRCA1/BRCA2/ATM (any source)	Deleterious in any of 15 HRR genes (any source)	Overall HRRm status
76	N	Y	Y	Y	Y	N	N	N	Y	N	N	N	N	Y			Partially characterized
77	N	Y	Y	Y	N	N	N	N	Y	Y	N	N	Y	Y			wt in plasma
78	N	Y	Y	Y	Y	N	N	N	Y	N	N	N	N	Y			Partially characterized
79	N	Y	Y	Y	Y	N	N	N	Y	Y	N	N	Y	Y			wt in plasma
80	N	Y	Y	Y	Y	N	N	N	Y	Y	N	N	Y	Y			wt in plasma
81	F	N	N	Y	Y	N	N	N	Y	Y	N	Y	N	Y			Partially characterized
82	N	N	N	N	N	N	N	N	N	N	N	N	N	N			Partially characterized
83	Y	N	N	Y	Y	N	N	N	Y	Y	N	N	Y	Y			wt in tissue
84	N	Y	Y	N	N	N	N	N	N	N	N	N	N	Y			Partially characterized
85	Y	N	N	Y	Y	N	N	N	Y	Y	N	N	Y	Y			wt in tissue
86	N	Y	Y	Y	Y	N	N	N	Y	Y	N	N	Y	Y		Y	Other HRRm (tail)
87	N	Y	Y	Y	Y	N	N	N	Y	Y	N	N	Y	Y		Y	Other HRRm (tail)
88	N	Y	Y	Y	Y	N	N	N	Y	N	N	N	N	Y			Partially characterized
89	N	Y	Y	Y	Y	N	N	N	Y	Y	N	N	Y	Y			wt in plasma

	Tumor	Germline		Plasma (ctDNA)											HRRm		
Patient no.	Tumor (FoundationOne)	Germline (Color Genomics)	Germline (HLI whole genome)	ctDNA (AZ100)	ctDNA LPWG	GH OMNI ctDNA result	RB ctDNA result	FMI ACT v2	ANY ctDNA Result	Evaluable ctDNA (high confidence somatic variant(s) in ctDNA NGS outputs)	ctDNA fraction ≤1%	ctDNA fraction between 1 and 5%	ctDNA fraction ≥5%	Data from at least one source	Deleterious in BRCA1/BRCA2/ATM (any source)	Deleterious in any of 15 HRR genes (any source)	Overall HRRm status
90	N	Y	Y	Y	Y	N	N	N	Y	Y	N	N	Y	Y			wt in plasma
91	N	Y	Y	Y	Y	N	N	N	Y	Y	N	N	Y	Y	Y	Y	BRCA/ATMm
92	N	Y	Y	N	N	Y	Y	N	Y	Y	Y	N	N	Y		Y	Other HRRm (tail)
93	N	Y	Y	Y	Y	N	N	N	Y	Y	N	Y	N	Y			Partially characterized
94	N	Y	Y	Y	Y	N	N	N	Y	N	N	N	N	Y			Partially characterized
95	F	Y	Y	Y	Y	N	N	N	Y	Y	N	N	Y	Y			wt in plasma
96	N	N	N	Y	Y	N	N	N	Y	Y	N	N	Y	Y			wt in plasma
97	N	N	N	Y	Y	N	N	N	Y	Y	N	N	Y	Y			wt in plasma
98	N	Y	Y	Y	Y	N	N	N	Y	Y	N	N	Y	Y			wt in plasma
99	N	Y	Y	Y	Y	N	N	N	Y	Y	N	N	Y	Y	Y	Y	BRCA/ATMm
100	N	Y	Y	Y	Y	N	N	N	Y	Y	N	N	Y	Y	Y	Y	BRCA/ATMm
101	N	Y	Y	Y	N	N	N	N	Y	Y	Y	N	N	Y	Y	Y	BRCA/ATMm
102	N	Y	Y	Y	Y	N	N	N	Y	Y	N	N	Y	Y			wt in plasma
103	N	Y	Y	Y	Y	N	N	N	Y	Y	N	N	Y	Y	Y	Y	BRCA/ATMm

	Tumor	Germline		Plasma (ctDNA)											HRRm		
Patient no.	Tumor (FoundationOne)	Germline (Color Genomics)	Germline (HLI whole genome)	ctDNA (AZ100)	ctDNA LPWG	GH OMNI ctDNA result	RB ctDNA result	FMI ACT v2	ANY ctDNA Result	Evaluable ctDNA (high confidence somatic variant(s) in ctDNA NGS outputs)	ctDNA fraction ≤1%	ctDNA fraction between 1 and 5%	ctDNA fraction ≥5%	Data from at least one source	Deleterious in BRCA1/BRCA2/ATM (any source)	Deleterious in any of 15 HRR genes (any source)	Overall HRRm status
104	N	Y	Y	Y	Y	N	N	N	Y	N	N	N	N	Y			Partially characterized
105	N	Y	Y	Y	Y	N	N	N	Y	Y	N	N	Y	Y	Y	Y	BRCA/ATMm
106	N	Y	Y	Y	Y	N	N	N	Y	Y	N	N	Y	Y			wt in plasma
107	N	Y	Y	Y	Y	N	N	N	Y	Y	N	Y	N	Y			Partially characterized
108	N	Y	Y	Y	Y	N	N	N	Y	Y	N	N	Y	Y			wt in plasma
109	N	Y	Y	F	N	N	N	N	N	N	N	N	N	Y			Partially characterized
110	N	Y	Y	Y	Y	N	N	N	Y	N	N	N	N	Y			Partially characterized
111	N	Y	Y	Y	Y	N	N	N	Y	Y	Y	N	N	Y			Partially characterized
112	N	Y	Y	Y	Y	N	N	N	Y	Y	N	N	Y	Y			wt in plasma
113	N	N	N	Y	Y	N	N	Y	Y	Y	N	N	Y	Y		Y	Other HRRm (tail)
114	N	N	N	Y	Y	N	N	N	Y	Y	N	N	Y	Y		Y	Other HRRm (tail)
115	Y	N	N	N	N	Y	Y	N	Y	Y	N	N	Y	Y			wt in tissue
116	Y	N	N	Y	Y	N	N	N	Y	Y	N	N	Y	Y			wt in tissue
117	N	Y	Y	Y	Y	N	N	N	Y	N	N	N	N	Y			Partially characterized

	Tumor	Germline		Plasma (ctDNA)											HRRm		
Patient no.	Tumor (FoundationOne)	Germline (Color Genomics)	Germline (HLI whole genome)	ctDNA (AZ100)	ctDNA LPWG	GH OMNI ctDNA result	RB ctDNA result	FMI ACT v2	ANY ctDNA Result	Evaluable ctDNA (high confidence somatic variant(s) in ctDNA NGS outputs)	ctDNA fraction ≤1%	ctDNA fraction between 1 and 5%	ctDNA fraction ≥5%	Data from at least one source	Deleterious in BRCA1/BRCA/ATM (any source)	Deleterious in any of 15 HRR genes (any source)	Overall HRRm status
118	N	Y	Y	Y	Y	N	N	N	Y	Y	N	N	Y	Y			wt in plasma
119	N	N	N	Y	Y	N	N	N	Y	Y	Y	N	N	Y			Partially characterized
120	F	Y	Y	Y	Y	N	N	N	Y	N	N	N	N	Y			Partially characterized
121	N	N	N	N	N	N	N	N	N	N	N	N	N	N			Partially characterized
122	N	Y	Y	Y	Y	N	N	N	Y	Y	N	N	Y	Y			wt in plasma
123	N	Y	Y	Y	Y	N	N	N	Y	Y	N	N	Y	Y			wt in plasma
124	N	N	N	N	N	N	N	N	N	N	N	N	N	N			Partially characterized
125	Y	Y	Y	Y	Y	N	N	N	Y	Y	N	N	Y	Y			wt in tissue
126	F	Y	Y	Y	Y	N	N	N	Y	Y	N	N	Y	Y			wt in plasma
127	F	Y	Y	Y	Y	N	N	N	Y	Y	N	N	Y	Y			wt in plasma
128	N	Y	Y	Y	Y	N	N	N	Y	Y	Y	N	N	Y			Partially characterized
129	Y	Y	Y	N	N	Y	Y	N	Y	Y	N	N	Y	Y			wt in tissue
130	F	Y	Y	Y	Y	N	N	N	Y	Y	N	N	Y	Y			wt in plasma
131	Y	Y	Y	N	N	Y	Y	N	Y	Y	N	Y	N	Y			wt in tissue

	Tumor	Germline		Plasma (ctDNA)											HRRm		
Patient no.	Tumor (FoundationOne)	Germline (Color Genomics)	Germline (HLI whole genome)	ctDNA (AZ100)	ctDNA LPWG	GH OMNI ctDNA result	RB ctDNA result	FMI ACT v2	ANY ctDNA Result	Evaluable ctDNA (high confidence somatic variant(s) in ctDNA NGS outputs)	ctDNA fraction ≤1%	ctDNA fraction between 1 and 5%	ctDNA fraction ≥5%	Data from at least one source	Deleterious in BRCA1/BRCA/ATM (any source)	Deleterious in any of 15 HRR genes (any source)	Overall HRRm status
132	Y	Y	Y	Y	Y	N	N	N	Y	Y	Y	N	N	Y			wt in tissue
133	F	Y	Y	Y	Y	N	N	Y	Y	Y	N	N	Y	Y			wt in plasma
134	Y	Y	Y	Y	Y	N	N	N	Y	Y	N	N	Y	Y			wt in tissue
135	F	Y	Y	Y	Y	N	N	N	Y	Y	N	Y	N	Y			Partially characterized
136	F	Y	Y	Y	Y	N	N	N	Y	Y	N	Y	N	Y	Y	Y	BRCA/ATMm
137	F	Y	Y	Y	Y	N	N	N	Y	N	N	N	N	Y			Partially characterized
138	F	N	N	Y	Y	N	N	N	Y	Y	N	Y	N	Y			Partially characterized
139	F	Y	Y	Y	Y	N	N	N	Y	Y	N	N	Y	Y			wt in plasma
140	Y	Y	Y	N	N	Y	Y	N	Y	Y	N	N	Y	Y			wt in tissue
141	N	Y	Y	Y	Y	N	N	N	Y	Y	N	N	Y	Y	Y	Y	BRCA/ATMm
142	Y	Y	Y	Y	Y	N	N	N	Y	Y	N	N	Y	Y			wt in tissue
YES	**38**	**102**	**102**	**110**	**103**	**18**	**17**	**5**	**129**	**101**	**9**	**16**	**76**	**136**	**14**	**23**	
NO	**74**	**40**	**40**	**26**	**39**	**122**	**121**	**137**	**13**	**41**				**6**			
FAIL	**30**	**0**	**0**	**6**	**0**	**2**	**4**	**0**	**0**	**0**				**0**			

ctDNA, circulating tumor DNA; FMI, Foundation Medicine Inc.; HLI, Human Longevity Inc.; HRRm, homologous recombination repair mutation; LPWG, low-pass whole genome; NGS, next-generation sequencing; wt, wild type

Table S3. All HRRm mutations

**Patient #** (assigned for this report)	**HRRm subgroup** (FINAL)*	**GERMLINE:** MUTATION DETECTED, not detected, NOT DONE, TECH FAIL†	**TISSUE:** MUTATION DETECTED, wt, NOT DONE, TECH FAIL†	**PLASMA:** MUTATION DETECTED, wt, not detected, NOT DONE, TECH FAIL†	**GENE**	**VARIANT**	**GERMLINE OR SOMATIC (if determined)**	**Plasma VAF (%) if reported‡**	**Plasma tumor fraction estimate** (ichorCNA or estimated from observed likely tumor VAFs or LPWG)	**COMMENT**
1	BRCA/ATMm	MUTATION DETECTED	TECH FAIL	TECH FAIL	*ATM*	DELETION exons62-63	GERMLINE	NA	NA	Confirmed by two germline assays (color and HLI WGS)
5	Other HRRm (tail)	MUTATION DETECTED	MUTATION DETECTED	Not detected	*CHEK2*	c.1100delC_p.T367fs*15	GERMLINE	NA	NA	External ctDNA assays masked CHEK2 region harboring this mutation (GH OMNI, ResBio ctDx-HRR)
10	Other HRRm (tail)	Not detected	TECH FAIL	MUTATION DETECTED	*BRIP1*	c.1727dupA_p.N576fs	SOMATIC	8.2	~14% (ichorCNA); 8% (VAFs)	Detected by AZ100
27	BRCA/ATMm	NOT DONE	TECH FAIL	MUTATION DETECTED	*ATM*	c.1608-2A>T (splice)	SOMATIC	7.8	~8% (VAFs)	Confirmed by two plasma assays (AZ100, FMI ACTv2)
33	Other HRRm (tail)	Not detected	wt	MUTATION DETECTED	*PPP2R2A*	DELETION (homozygous)	SOMATIC	NA	~75% (ichorCNA); 60% (VAFs)	Detected by AZ100, confirmed in low-pass whole genome
46	BRCA/ATMm	NOT DONE	MUTATION DETECTED	Not detected	*BRCA2*	c.3860_3861insA_p.N1287fs*2	SOMATIC	ND	<1% (low/non-shedder)	13% VAF in tumor; low tumor fraction/non-shedder plasma samples
56	BRCA/ATMm	NOT DONE	TECH FAIL	MUTATION DETECTED	*ATM x2*	c.2548G>T_p.E850* and c.3446delA_p.N1149fs	2X SOMATIC	17, 15	~30% (ichorCNA)	Confirmed by two plasma assays (AZ100, FMI ACTv2)
65	Other HRRm (tail)	MUTATION DETECTED	NOT DONE	MUTATION DETECTED	*CHEK2*	c.1100delC_p.T367fs*15	GERMLINE	NA	~1% (VAFs), low shedder	External ctDNA assay masked CHEK2 region harboring this mutation (ResBio ctDx-HRR)
66	BRCA/ATMm	MUTATION DETECTED	MUTATION DETECTED	MUTATION DETECTED	*ATM*	c.3802delG_p.V1268fs*1	GERMLINE	NA	~20% (ichorCNA)	
71	BRCA/ATMm	Not detected	NOT DONE	MUTATION DETECTED	*BRCA2*	c.1965_1974delAACTTTGTCC_p.T656fs	SOMATIC	21	~19% (ichorCNA)	
86	Other HRRm (tail)	Not detected	NOT DONE	MUTATION DETECTED	*CHEK1*	DELETION exon11 (homozygous)	SOMATIC	NA	~52% (ichorCNA)	
87	Other HRRm (tail)	Not detected	NOT DONE	MUTATION DETECTED	*CDK12 x2*	c.1257_1264delGTCCAAGGinsA_p.S420fs and c.452C>G_p.S151*	2X SOMATIC	8.8, 3	~9% (VAFs)	

**Patient #** (assigned for this report)	**HRRm subgroup** (FINAL)*	**GERMLINE:** MUTATION DETECTED, not detected, NOT DONE, TECH FAIL†	**TISSUE:** MUTATION DETECTED, wt, NOT DONE, TECH FAIL†	**PLASMA:** MUTATION DETECTED, wt, not detected, NOT DONE, TECH FAIL†	**GENE**	**VARIANT**	**GERMLINE OR SOMATIC (if determined)**	**Plasma VAF (%) if reported‡**	**Plasma tumor fraction estimate** (ichorCNA or estimated from observed likely tumor VAFs or LPWG)	**COMMENT**
91	BRCA/ATMm	MUTATION DETECTED	NOT DONE	MUTATION DETECTED	*BRCA2 x2*	c.2806_2809delAAAC_p.A938Pfs*21 and c.1837delC_p.Leu613fs	GERMLINE & SOMATIC	32	~39% (ichorCNA); ~32% (VAFs)	Both events seen in plasma assay
92	Other HRRm (tail)	MUTATION DETECTED	NOT DONE	MUTATION DETECTED	*PALB2*	c.1592delT_p.L531Cfs*30	GERMLINE	NA	NA	
99	BRCA/ATMm	Not detected	NOT DONE	MUTATION DETECTED	*BRCA2*	DELETION (homozygous)	SOMATIC		~47% (ichorCNA); ~40% (VAFs)	Detected in ctDNA low-pass whole genome, confirmed in ctDNA AZ100
100	BRCA/ATMm	Not detected	NOT DONE	MUTATION DETECTED	*BRCA2*	c.8755-1G>T (splice)	SOMATIC	0.58	~7% (VAFs)	
101	BRCA/ATMm	Not detected	NOT DONE	MUTATION DETECTED	*BRCA2*	c.2918C>A_p.S973*	SOMATIC	0.65	<1% (low/non-shedder)	
103	BRCA/ATMm	Not detected	NOT DONE	MUTATION DETECTED	*BRCA2*	c.7015A>T_p.K2339*	SOMATIC	0.57	~6% (VAFs)	
105	BRCA/ATMm	Not detected	NOT DONE	MUTATION DETECTED	*ATM*	DELETION exon4 (homozygous)	SOMATIC	NA	~49% (ichorCNA); ~39% (VAFs)	
113	Other HRRm (tail)	NOT DONE	NOT DONE	MUTATION DETECTED	*CDK12*	c.2797A>T_p.K933*	SOMATIC	32	~71% (ichorCNA); ~32% (VAFs)	Confirmed by two plasma assays (AZ100, FMI ACTv2)
114	Other HRRm (tail)	NOT DONE	NOT DONE	MUTATION DETECTED	*CDK12*	c.2343_2350delAATCCACC_p.I782fs	SOMATIC	6.9	~7% (VAFs)	
136	BRCA/ATMm	Not detected	TECH FAIL	MUTATION DETECTED	*ATM*	c.5618_5630delGTCTTCGACACTT_p.C1873fs	SOMATIC	2.09	~2% (VAFs)	
141	BRCA/ATMm	MUTATION DETECTED	NOT DONE	MUTATION DETECTED	*ATM*	c.4804_4805delGT_p.V1602Lfs*2	GERMLINE	NA	~24% (ichorCNA); ~25% (VAFs)	

*BRCA/ATMm, Deleterious alteration in *BRCA1*, *BRCA2* or *ATM* detected; Other HRRm (tail), deleterious alteration in one of 12 other HRR genes (*BARD1*, *BRIP1*, *CDK12*, *CHEK1*, *CHEK2*, *FANCL*, *PALB2*, *PPP2R2A*, *RAD51B*, *RAD51C*, *RAD51D*, *RAD54L*); wt in tissue, wildtype (no deleterious alteration in any HRR gene was detected in FMI tumor tissue test); unknown, HRR status unknown (includes: patients without sample for analysis; patients whose samples failed testing; patients who had a germline and/or plasma result with no HRR alteration detected which is not a definitive result and therefore cannot be classed as wt). †m, mutated (subject carries a deleterious alteration in the HRR gene listed); wt, wildtype (no deleterious alteration in any HRR gene was detected in FMI tumor tissue test); not detected, no deleterious HRR gene alteration was detected in germline and/or plasma testing; NOT DONE, no sample was available for analysis, or analysis was not required because biomarker status was already determined by another method; TECH FAIL, sample was available, but the sample failed QC prior to or after sequencing, thus no results were generated. ‡Frequency of small variants reported in one or more ctDNA assays when variants considered somatic; NA, not applicable (variant was germline or a deletion); ND, not detected (very low ctDNA fraction/non-shedder).

ctDNA, circulating tumor DNA; FMI, Foundation Medicine Inc.; HRRm, homologous recombination repair mutation; LPWG, low-pass whole genome; wt, wild type; VAF, variant allele frequency.

Table S4. Metrics for all plasma samples analyzed in-house

Patient # (assigned for this manuscript)	Clinical site	Plasma volume (mL)	ctDNA mass (ng)	Mass used (ng)	PCR cycles, n	Library mean size (bp)	Library mass (ng)	Reads	% mapped reads	% Duplicate reads	% on target	% useable	Mean unique depth	Error rate, %	%GC	Insert size (bp)
4	1	1.9	32.16	32.16	9	509	102.69	16.28 M	87.8	58.6	72.8	64.8	2667	0.57	48	226
7	1	1.2	54.26	54.26	5	408	244.8	50.22 M	81.7	63.6	81.1	67.1	8796	0.57	48	182
8	1	1.6	184.45	150.00	4	418	135.24	29.71 M	81.5	64.3	81.2	67.2	5183	0.55	47	184
9	2	1.7	3.68	3.68	10	388	709.8	5.17 M	90.0	50.0	81.6	74.8	1008	0.46	50	174
10	2	1.7	12.94	12.94	7	387	319.2	11.30 M	93.3	53.7	85.1	80.7	2399	0.39	49	170
12	3	1.9	21.72	21.72	8	379	907.2	15.99 M	88.8	57.5	80.8	73.1	3007	0.52	51	175
14	3	1.7	16.54	16.54	7	367	415.8	12.32 M	92.9	53.6	86.9	82.1	2693	0.34	49	169
16	4	1.8	4.41	4.41	10	370	261.8	5.71 M	82.2	54.2	78.0	65.2	968	0.57	49	173
19	5	0.85	17.86	17.86	8	403	409.2	16.85 M	90.2	55.7	80.9	74.2	3262	0.44	48	174
22	5	0.9	8.84	8.84	7	413	165.2	15.43 M	87.6	53.4	74.5	66.3	2586	0.68	47	172
23	5	1.15	4.87	4.87	8	426	234.3	6.82 M	75.8	49.1	67.8	52.1	891	0.91	47	174
26	5	1.25	32.12	32.12	7	431	216.48	23.18 M	91.1	54.2	81.7	75.6	4568	0.42	47	177
27	5	1.5	12.55	12.55	7	401	320.8	15.35 M	87.3	53.3	73.9	65.6	2547	0.69	48	173
28	6	1.6	18.17	18.17	7	425	496	15.32 M	91.7	55.6	83.8	78.0	3064	0.39	47	172
29	7	1.7	27.14	27.14	6	397	448	17.54 M	93.8	52.7	86.8	82.4	3850	0.40	48	175
30	7	1.7	10.21	10.21	8	403	562	10.18 M	91.8	50.3	77.9	72.6	1864	0.41	48	171
33	8	1.6	130.82	130.82	5	399	917.7	39.64 M	83.3	62.5	82.1	69.3	7193	0.67	48	183
34	9	1.4	20.67	20.67	6	414	310.5	18.64 M	83.6	61.3	79.3	67.2	3214	0.55	48	176

Patient # (assigned for this manuscript)	Clinical site	Plasma volume (mL)	ctDNA mass (ng)	Mass used (ng)	PCR cycles, n	Library mean size (bp)	Library mass (ng)	Reads	% mapped reads	% Duplicate reads	% on target	% useable	Mean unique depth	Error rate, %	%GC	Insert size (bp)
35	9	1.1	17.52	17.52	8	385	466.2	16.10 M	89.3	60.0	78.9	71.6	2943	0.54	49	181
36	9	1.15	5.48	5.48	8	394	403.2	5.06 M	89.6	52.0	83.7	76.3	1005	0.47	48	172
37	9	1.4	18.64	18.64	7	395	288	10.33 M	93.1	54.8	86.6	82.0	2219	0.34	48	167
39	9	1.55	3.74	3.74	8	397	297.75	6.81 M	76.2	50.9	69.6	53.9	924	0.85	48	173
40	9	2	17.98	17.98	7	425	430	11.68 M	92.4	54.7	83.8	78.7	2373	0.41	48	175
42	9	1.7	42.50	42.50	5	411	577.5	23.98 M	92.6	55.4	86.2	81.1	5165	0.59	48	176
43	10	1.8	8.89	8.89	10	401	129.57	1.56 M	83.4	31.5	56.3	48.0	189	0.66	50	169
45	11	1	27.47	27.47	6	398	317.1	13.04 M	93.6	53.6	86.5	82.2	2841	0.45	48	172
46	11	1.6	19.58	19.58	8	399	703.5	18.18 M	91.2	56.3	80.0	74.2	3438	0.48	49	175
47	11	1.3	5.97	5.97	8	403	533.4	6.68 M	88.7	57.0	84.8	76.5	1338	0.52	49	172
48	12	1.3	3.14	3.14	8	422	396.9	2.86 M	87.6	43.9	77.1	68.6	502	0.53	48	178
49	12	1.9	14.58	14.58	7	400	361.2	10.49 M	94.0	48.4	80.8	77.3	2112	0.49	49	173
51	12	1.8	21.12	21.12	8	372	600.6	20.34 M	89.8	58.2	80.1	73.0	3791	0.48	49	175
52	12	1.8	25.11	25.11	6	398	279.3	11.21 M	93.2	54.3	86.0	81.4	2403	0.46	48	172
53	13	1.3	5.70	5.70	8	403	472.5	6.93 M	89.7	54.0	82.9	75.5	1358	0.46	48	174
54	13	1.7	6.31	6.31	10	389	625.8	10.17 M	88.9	54.8	79.5	71.8	1855	0.46	48	172
55	13	1.45	9.12	9.12	7	433	281.45	12.57 M	86.4	53.5	69.5	61.3	1943	0.74	48	176
56	13	2.1	20.99	20.99	10	458	422.1	18.34 M	86.2	55.9	73.1	63.9	2971	0.55	48	186
59	14	1.6	189.24	150.00	5	382	690.9	40.51 M	91.3	57.6	88.3	81.9	8856	0.59	47	169

Patient # (assigned for this manuscript)	Clinical site	Plasma volume (mL)	ctDNA mass (ng)	Mass used (ng)	PCR cycles, n	Library mean size (bp)	Library mass (ng)	Reads	% mapped reads	% Duplicate reads	% on target	% useable	Mean unique depth	Error rate, %	%GC	Insert size (bp)
60	15	1.65	10.71	10.71	7	396	499.8	8.53 M	94.2	44.5	79.4	76.2	1685	0.46	49	172
61	15	0.85	21.66	21.66	6	387	424.2	11.67 M	93.8	55.4	84.9	81.0	2480	0.44	48	170
62	16	0.7	5.61	5.61	8	389	136.15	7.99 M	85.1	44.3	64.2	55.6	1124	0.75	49	170
63	17	1.25	21.96	21.96	7	381	458	12.43 M	92.3	53.8	86.7	81.2	2684	0.39	47	170
64	17	1.4	5.00	5.00	10	416	701.4	7.61 M	88.4	50.6	78.0	70.2	1349	0.49	49	176
65	18	1.7	10.55	10.55	9	390	642.6	11.99 M	90.0	54.6	76.8	70.3	2122	0.52	48	178
66	18	1.8	16.44	16.44	8	376	651	21.26 M	89.2	58.2	82.0	74.3	4075	0.50	48	172
67	19	1.9	5.86	5.86	8	409	459.9	5.83 M	89.6	53.3	82.7	75.4	1137	0.50	49	174
68	19	1.9	16.52	16.52	6	405	359.1	10.62 M	93.6	53.5	83.6	79.6	2206	0.46	49	176
69	19	1.1	20.90	20.90	6	419	541.8	14.68 M	93.5	52.7	82.1	77.9	2965	0.47	48	180
70	19	2	45.12	45.12	5	398	714	24.38 M	92.5	60.0	86.4	81.2	5268	0.62	49	175
71	19	2.2	8.65	8.65	9	421	629.2	9.85 M	83.8	56.2	79.2	67.5	1719	0.53	48	172
72	19	1.4	7.05	7.05	9	488	98.7	2.95 M	88.5	37.1	67.8	61.0	451	0.54	47	174
73	19	1.8	7.61	7.61	8	421	352	10.39 M	91.9	50.2	76.0	71.0	1859	0.43	49	173
74	19	2.1	383.18	150.00	4	441	486.45	20.49 M	91.8	57.2	85.2	79.7	4357	0.45	48	182
75	19	1.9	13.67	13.67	8	434	428.4	17.50 M	90.1	55.4	75.6	69.3	3055	0.51	49	183
76	19	2.4	11.79	11.79	8	452	144.29	4.79 M	91.6	46.5	79.0	73.6	903	0.42	49	174
77	19	2.1	10.79	10.79	8	400	70.97	2.18 M	92.4	39.6	78.9	74.2	427	0.38	49	170
78	19	1.9	13.48	13.48	7	397	348.6	12.76 M	93.0	54.3	85.1	80.4	2701	0.40	49	173

Patient # (assigned for this manuscript)	Clinical site	Plasma volume (mL)	ctDNA mass (ng)	Mass used (ng)	PCR cycles, n	Library mean size (bp)	Library mass (ng)	Reads	% mapped reads	% Duplicate reads	% on target	% useable	Mean unique depth	Error rate, %	%GC	Insert size (bp)
79	19	1.6	15.23	15.23	7	418	221.37	7.87 M	92.5	54.7	83.9	78.9	1609	0.38	49	171
80	19	1.9	146.94	146.94	4	418	418	61.92 M	82.0	61.8	80.8	67.1	10903	0.58	48	185
81	20	1.45	3.70	3.70	8	430	279.5	7.83 M	76.5	52.6	68.7	53.4	1047	0.80	47	176
83	20	1.5	14.28	14.28	8	396	514.5	13.75 M	89.6	57.8	78.3	71.2	2485	0.51	48	181
85	20	1.7	13.58	13.58	8	405	430.5	14.47 M	89.4	57.4	77.3	70.2	2569	0.54	48	184
86	21	1.7	290.16	150.00	5	357	1113	33.27 M	89.7	62.5	89.7	81.5	7395	0.39	50	165
87	21	1.1	13.35	13.35	8	378	430	7.52 M	93.3	48.9	83.2	78.9	1552	0.37	48	172
88	21	1.8	26.36	26.36	7	386	472.35	15.64 M	91.8	62.2	87.9	82.0	3431	0.41	49	172
89	21	1.5	41.85	41.85	6	435	265.55	15.27 M	93.2	54.9	85.0	80.5	3257	0.40	47	176
90	21	1.75	27.06	27.06	7	419	361.9	13.74 M	92.0	60.3	86.4	80.7	2926	0.40	48	174
91	21	1.8	99.48	99.48	5	412	852.6	39.45 M	84.4	63.2	80.9	69.1	7065	0.63	47	187
93	21	1.85	20.18	20.18	8	368	460	8.76 M	92.8	49.4	82.0	77.4	1763	0.39	48	178
94	21	2.4	25.87	25.87	7	378	440	13.20 M	91.7	56.0	84.2	78.4	2709	0.43	48	181
95	21	1.1	23.33	23.33	7	366	520.8	14.69 M	91.3	54.5	87.2	81.0	3165	0.34	48	163
96	21	2.2	40.01	40.01	5	415	646.8	21.88 M	92.8	54.3	85.1	80.2	4621	0.57	49	179
97	21	1.4	19.27	19.27	6	380	495.6	12.50 M	94.0	52.2	85.3	81.6	2702	0.44	49	168
98	22	1.6	10.38	10.38	7	408	611.1	15.10 M	93.8	48.5	79.8	76.1	2980	0.50	48	174
99	22	2	36.94	36.94	7	431	741.4	31.58 M	88.7	58.9	82.9	74.7	6177	0.53	48	182
100	22	2.05	6.20	6.20	9	420	429	10.03 M	81.1	57.9	77.3	63.7	1651	0.62	48	174

Patient # (assigned for this manuscript)	Clinical site	Plasma volume (mL)	ctDNA mass (ng)	Mass used (ng)	PCR cycles, n	Library mean size (bp)	Library mass (ng)	Reads	% mapped reads	% Duplicate reads	% on target	% useable	Mean unique depth	Error rate, %	%GC	Insert size (bp)
101	22	2	7.12	7.12	7	467	169.18	8.40 M	82.2	56.5	77.4	64.6	1390	0.61	49	189
102	22	1.4	7.83	7.83	7	379	363.3	9.58 M	94.2	43.6	78.7	75.5	1883	0.48	49	168
103	22	2	18.72	18.72	8	408	299.2	13.72 M	91.2	55.3	82.3	76.2	2727	0.46	49	178
104	22	1.4	7.38	7.38	7	401	405.3	9.68 M	93.8	44.4	75.7	72.3	1808	0.46	48	170
105	22	1.3	113.40	113.40	5	389	1234.8	30.85 M	88.0	60.8	87.3	78.0	6422	0.42	49	172
106	23	1.6	3.32	3.32	10	421	409.5	5.17 M	89.6	46.4	79.6	72.5	972	0.46	48	173
107	23	1.8	14.93	14.93	7	384	352.8	11.63 M	92.8	53.4	85.3	80.4	2472	0.34	48	171
108	23	1.5	31.06	31.06	6	422	319.6	16.24 M	94.0	54.8	86.0	82.1	3553	0.38	48	175
110	23	1.1	4.58	4.58	10	425	627.9	5.43 M	89.1	49.3	80.6	72.9	1024	0.48	48	177
111	23	1.8	8.37	8.37	8	416	840	15.10 M	92.8	54.5	83.8	79.1	3122	0.42	48	176
112	23	1.5	53.30	53.30	6	364	783.3	23.51 M	89.1	61.2	88.6	80.3	5075	0.39	48	166
113	24	1.9	70.47	70.47	5	409	1587.6	33.76 M	92.9	58.6	86.0	81.4	7318	0.60	48	176
114	24	2.1	7.19	7.19	7	444	344.4	7.34 M	93.5	40.4	73.7	70.1	1314	0.50	49	179
116	24	1.6	4.83	4.83	10	385	480.9	5.45 M	87.4	42.8	73.5	65.6	910	0.55	51	171
117	25	1.1	7.27	7.27	8	388	338.1	6.61 M	90.3	52.8	84.5	77.5	1344	0.42	48	172
118	25	1	35.51	35.51	6	387	584	23.62 M	89.8	57.2	85.1	77.6	4759	0.42	47	171
119	25	1.2	3.58	3.58	8	395	539.7	4.15 M	89.3	49.1	82.0	74.5	803	0.47	48	172
120	25	1.4	27.37	27.37	7	405	310.2	14.40 M	93.7	54.5	86.9	82.6	3173	0.38	48	172
122	25	1	132.44	132.44	5	377	900.9	23.79 M	88.4	59.7	88.0	78.9	5033	0.41	48	169

Patient # (assigned for this manuscript)	Clinical site	Plasma volume (mL)	ctDNA mass (ng)	Mass used (ng)	PCR cycles, n	Library mean size (bp)	Library mass (ng)	Reads	% mapped reads	% Duplicate reads	% on target	% useable	Mean unique depth	Error rate, %	%GC	Insert size (bp)
123	25	0.9	7.54	7.54	8	404	216.3	5.01 M	90.1	48.5	80.9	74.0	961	0.43	48	176
125	26	1.5	650.00	150.00	5	367	947.1	32.97 M	81.6	67.1	83.9	69.5	6022	0.64	49	174
126	26	0.9	5.07	5.07	10	386	510.3	4.94 M	88.9	48.6	81.1	73.3	951	0.45	48	171
127	26	0.8	16.80	16.80	7	375	365.4	10.59 M	92.4	54.0	85.8	80.7	2270	0.34	48	168
128	26	1.7	10.86	10.86	8	390	294	6.44 M	93.2	50.2	82.3	78.0	1308	0.38	49	174
130	26	1.2	17.63	17.63	7	420	375.9	10.63 M	92.0	53.4	83.0	77.6	2162	0.37	48	177
132	26	0.9	15.04	15.04	8	420	264	12.07 M	91.0	55.7	85.8	79.4	2548	0.39	49	167
133	26	1.7	134.46	134.46	6	386	1407	23.60 M	87.8	61.7	87.5	78.0	4945	0.44	49	171
134	26	1.7	10.29	10.29	9	394	627.9	15.15 M	88.7	59.9	83.8	75.6	2944	0.49	49	175
135	26	1.6	11.12	11.12	8	397	474.6	8.40 M	93.8	47.5	82.9	79.1	1726	0.37	48	174
136	26	1.3	9.86	9.86	8	395	344.4	7.58 M	92.9	48.4	83.0	78.4	1550	0.39	48	172
137	27	1.6	12.38	12.38	7	403	358	10.24 M	94.2	53.0	85.7	82.1	2191	0.33	49	168
138	28	1.55	20.50	20.50	6	469	375.2	25.46 M	84.8	58.9	78.5	67.5	4397	0.54	48	181
139	29	2.2	22.03	22.03	7	387	667.8	14.63 M	91.7	55.6	85.8	80.1	3114	0.40	49	172
141	30	1.3	13.94	13.94	8	396	728.7	16.36 M	88.5	58.1	80.5	72.6	3058	0.63	50	175
142	31	1.5	28.81	28.81	8	402	1041.6	30.10 M	88.6	58.2	79.8	71.8	5537	0.52	48	178

ctDNA, circulating tumor DNA; PCR, polymerase chain reaction; %GC, percentage GC base composition

Table S5. HRRm concordance between (A) tissue vs plasma, and (B) germline vs plasma

(A)

		Tissue HRRm		
		Not detected (–)	Detected (+)	TOTAL
Plasma HRRm	Not detected (–)	33	1[a]	34
	Detected (+)	0	2	2
	TOTAL	33	3	36
		NPA: 33/33 (100%)	PPA: 2/3 (67%)	

(B)

		Germline HRRm		
		Not detected (–)	Detected (+)	TOTAL
Plasma HRRm	Not detected (–)	89	0	89
	Suspected germline detected[b] (+)	0	6	6
	TOTAL	89	6	95
		NPA: 89/89 (100%)	PPA: 6/6 (100%)	

NPA was defined as the number of patients labelled HRR mutation negative based on a tissue or germline test who remained HRR negative with a plasma test. PPA was defined as the number of patients labelled HRR mutation positive based on a tissue or germline test and who were also called positive in a plasma test.HRR, homologous recombination repair; HRRm, HRR mutation; NPA, negative percent agreement; PPA, positive percent agreement; VAF, variant allele frequency. [a]Tumor positive (HRRm) plasma negative discordant. This case had no high confidence somatic alterations detected in ctDNA and was classed as a non-shedder. [b]HRRm observed at or near 50% or 100% VAF, strongly suggestive of germline origin.

Figure S1. Plots illustrating (A) mass of ctDNA, and (B) volumes of plasma obtained per 2 × 1 mL aliquots of plasma provided by clinical site

(A)

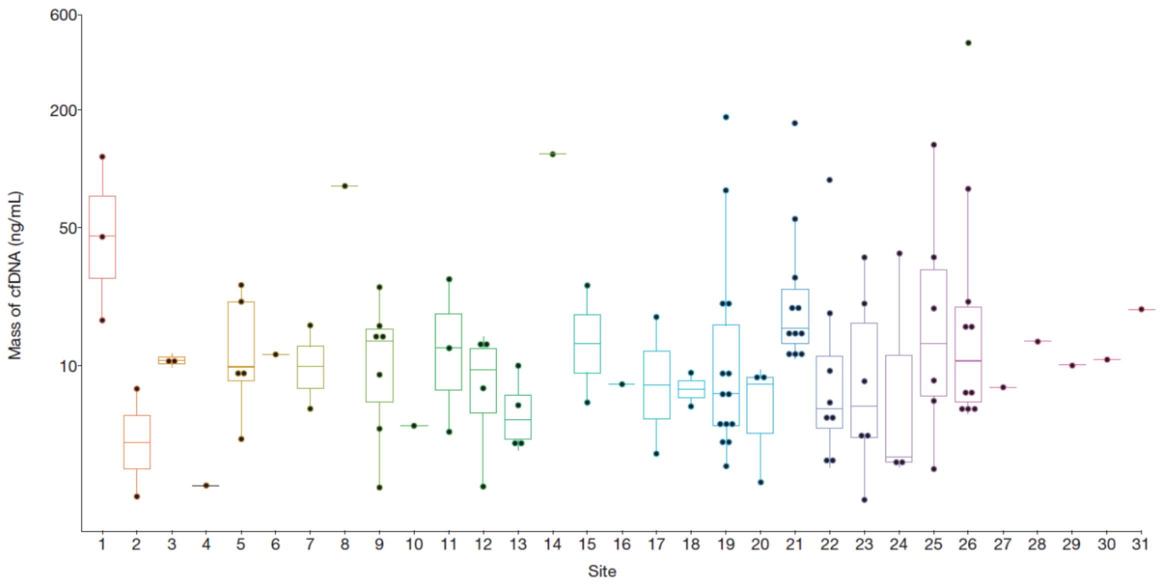

(B)

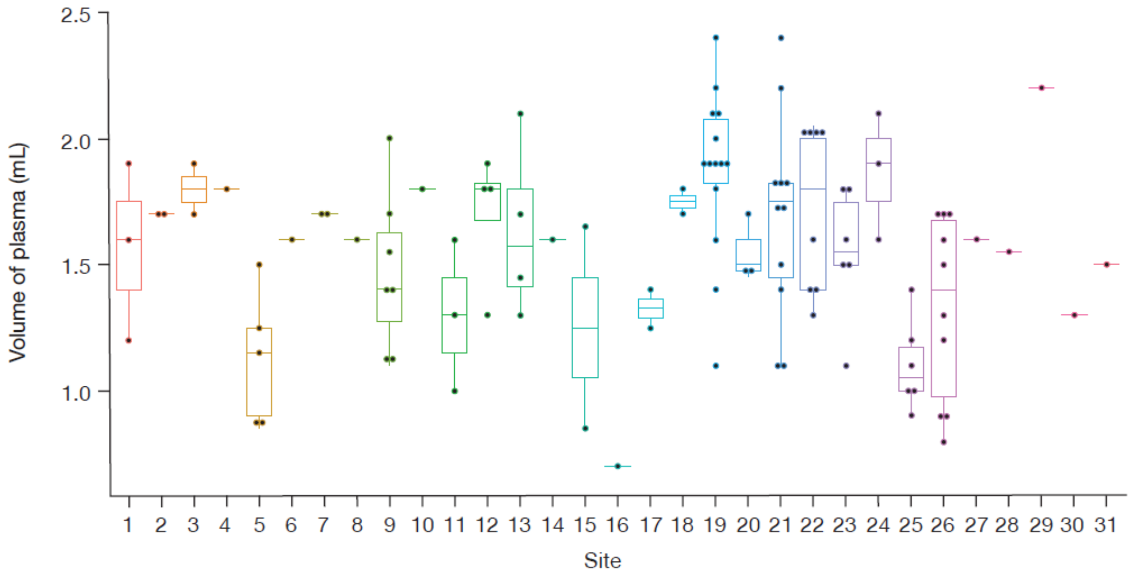

ctDNA, circulating tumor DNA

Figure S2. Median unique read coverage of key HRR genes across all samples analyzed via AZ100 assay

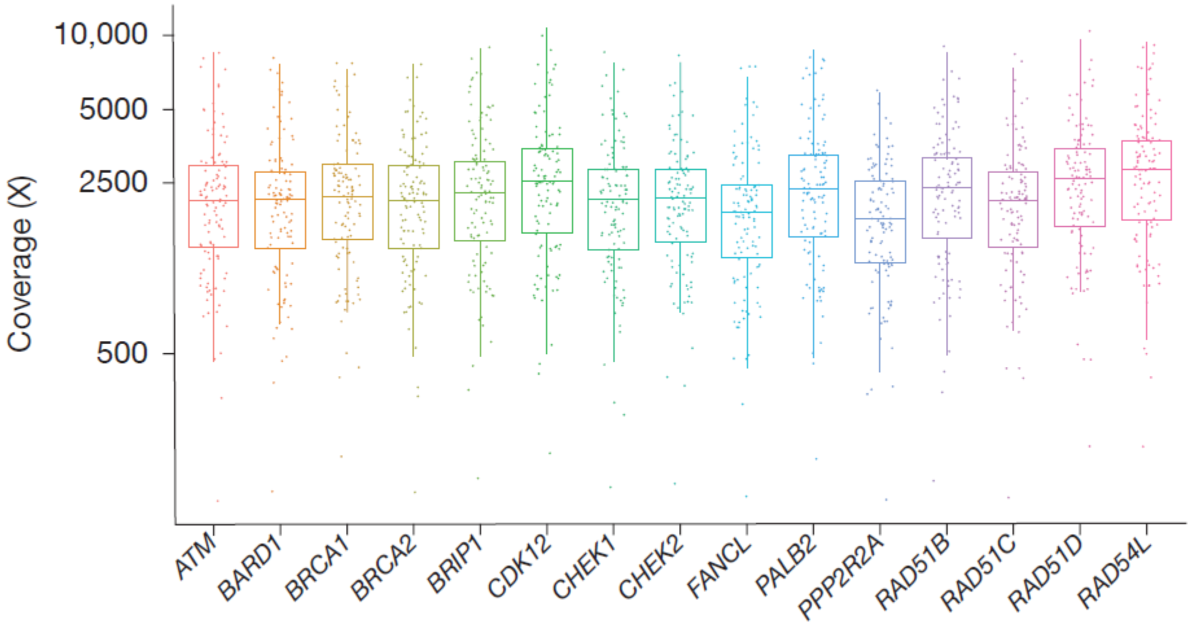

HRR, homologous recombination repair

Figure S3. Visualization of LPWG data from ctDNA libraries. (**A**) Heatmap of Log2 Ratios for HMMCopy data from LPWG sequencing for all ctDNA libraries successfully sequenced in-house. Only data covering the listed subset of genes is shown. Red and blue represent likely copy loss and gain/amplification, respectively. Green bars indicate the ctDNA fraction as estimated by ichorCNA. Samples are clustered on the similarity of the log2 values per patient using the Euclidian distance measure. *AR* amplification is highly prevalent and more easily detected in samples with higher apparent ctDNA fraction. *PTEN* deletion is also prevalent and more apparent in samples with higher ctDNA fraction but with lower sensitivity than for *AR* amplification. A potential deep (homozygous) deletion in *BRCA2* is highlighted (patient 99). (**B**) Visualization of the deep *BRCA2* deletion in the same data for patient 99 across the genome and chr13. Coloring of data points based on deviation of Log2 ratio from expected: pale blue – approximately neutral, green – low level gain, yellow – high level gain/amplification, red – deletion. IchorCNA calculated the tumor fraction in this sample as 47%, matching well with other mutations seen in targeted data. There is clear evidence of a large single copy loss across much of chr13 and an additional focal loss at the *BRCA2* locus. We observed no evidence of LoH in our targeted data (for SNPs covered in and close to the *BRCA2* gene) strongly suggesting true homozygous loss in the tumor.

(A)

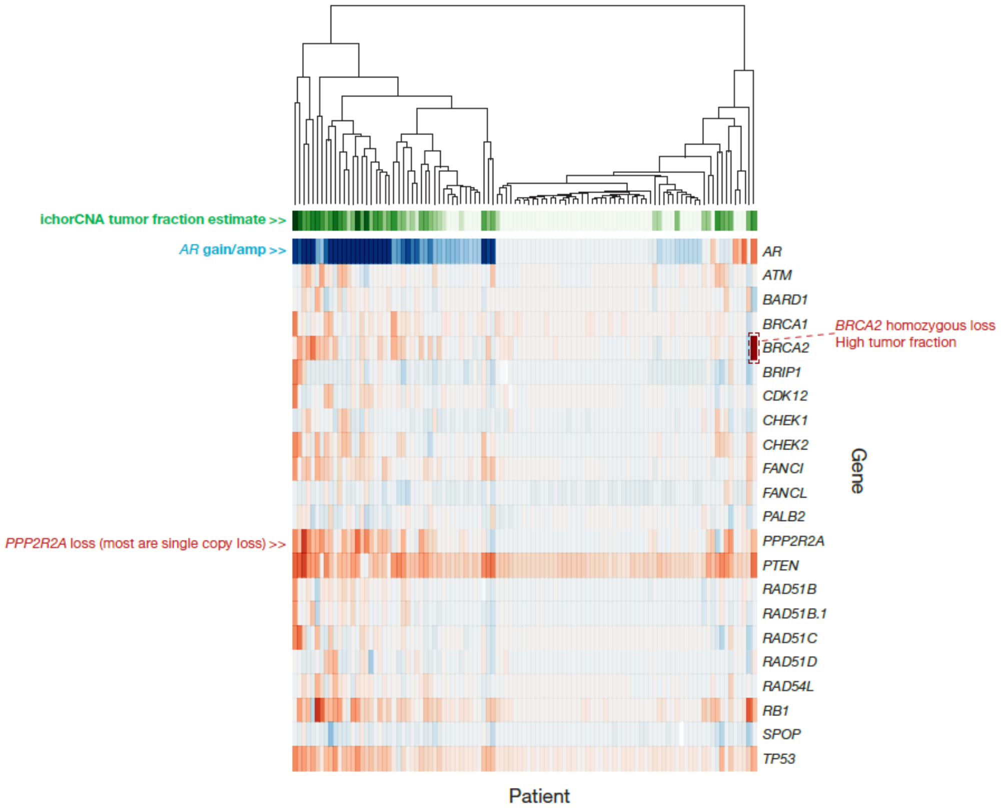

(B)

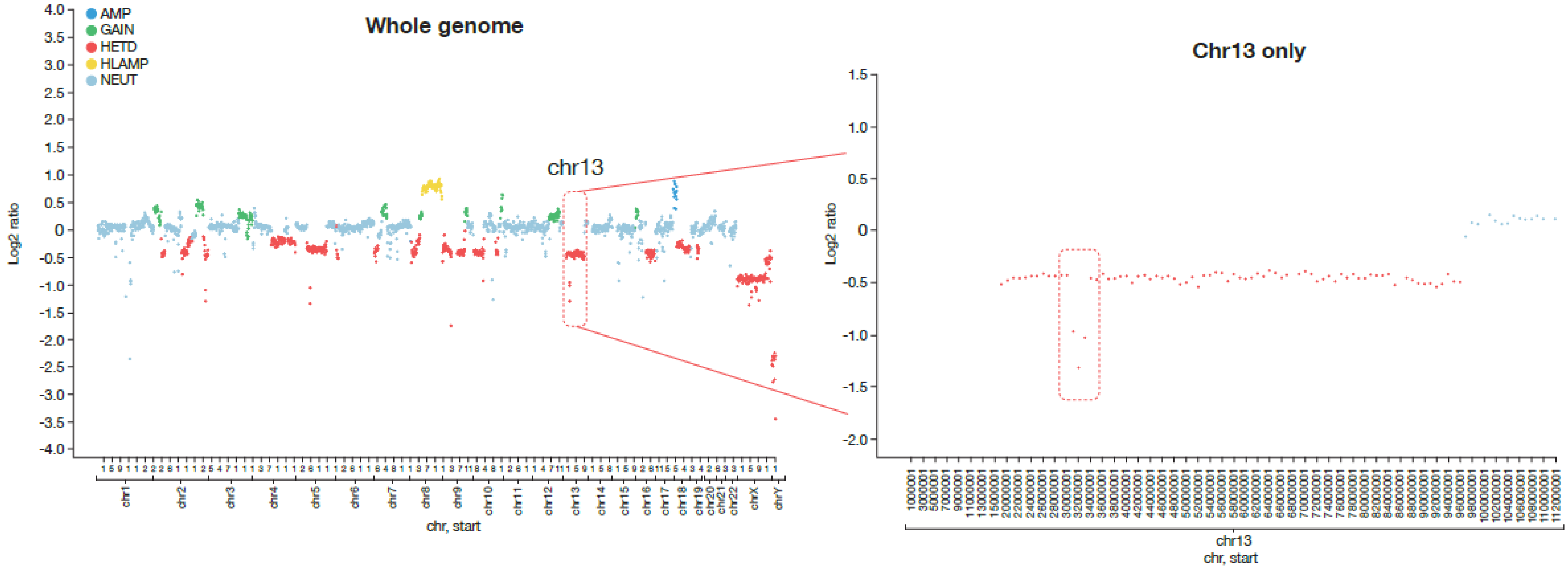

ctDNA, circulating tumor DNA; LPWG, low-pass whole genome; LoH, loss of heterozygosity; SNP, single nucleotide polymorphism

SUPPLEMENTAL REFERENCES

1. Clarke, N.; Wiechno, P.; Alekseev, B.; Sala, N.; Jones, R.; Kocak, I.; Chiuri, V.E.; Jassem, J.; Flechon, A.; Redfern, C.; et al. Olaparib combined with abiraterone in patients with metastatic castration-resistant prostate cancer: a randomised, double-blind, placebo-controlled, phase 2 trial. *Lancet Oncol.* **2018**, *19*, 975-986, doi:10.1016/S1470-2045(18)30365-6.
2. Rowlands, V.; Rutkowski, A.J.; Meuser, E.; Carr, T.H.; Harrington, E.A.; Barrett, J.C. Optimisation of robust singleplex and multiplex droplet digital PCR assays for high confidence mutation detection in circulating tumour DNA. *Sci. Rep.* **2019**, *9*, 12620, doi:10.1038/s41598-019-49043-x.
3. bcbio. Available online: https://github.com/bcbio/bcbio-nextgen (accessed on 21 February 2010).
4. Li, H.; Durbin, R. Fast and accurate short read alignment with Burrows-Wheeler transform. *Bioinformatics* **2009**, *25*, 1754-1760, doi:10.1093/bioinformatics/btp324.
5. Ewels, P.; Magnusson, M.; Lundin, S.; Kaller, M. MultiQC: summarize analysis results for multiple tools and samples in a single report. *Bioinformatics* **2016**, *32*, 3047-3048, doi:10.1093/bioinformatics/btw354.
6. fgbio. Available online: https://github.com/fulcrumgenomics/fgbio (accessed on 21 February 2020).
7. Lai, Z.; Markovets, A.; Ahdesmaki, M.; Chapman, B.; Hofmann, O.; McEwen, R.; Johnson, J.; Dougherty, B.; Barrett, J.C.; Dry, J.R. VarDict: a novel and versatile variant caller for next-generation sequencing in cancer research. *Nucleic Acids Res.* **2016**, *44*, e108, doi:10.1093/nar/gkw227.
8. Cingolani, P.; Platts, A.; Wang le, L.; Coon, M.; Nguyen, T.; Wang, L.; Land, S.J.; Lu, X.; Ruden, D.M. A program for annotating and predicting the effects of single nucleotide polymorphisms, SnpEff: SNPs in the genome of Drosophila melanogaster strain w1118; iso-2; iso-3. *Fly (Austin)* **2012**, *6*, 80-92, doi:10.4161/fly.19695.
9. Lai, Z. Seq2C. Available online: https://github.com/AstraZeneca-NGS/Seq2C (accessed on 21 February 2020).
10. Epam Systems. New genome browser - NGB. Available online: https://lifescience.opensource.epam.com/ngb/index.html (accessed on January 4th 2021)
11. DKFZBiasFilter. Available online: https://github.com/DKFZ-ODCF/DKFZBiasFilter (accessed on 21 February 2020).
12. Lek, M.; Karczewski, K.J.; Minikel, E.V.; Samocha, K.E.; Banks, E.; Fennell, T.; O'Donnell-Luria, A.H.; Ware, J.S.; Hill, A.J.; Cummings, B.B.; et al. Analysis of protein-coding genetic variation in 60,706 humans. *Nature* **2016**, *536*, 285-291, doi:10.1038/nature19057.
13. Karczewski, K.J.; Francioli, L.C.; Tiao, G.; Cummings, B.B.; Alföldi, J.; Wang, Q.; Collins, R.L.; Laricchia, K.M.; Ganna, A.; Birnbaum, D.P.; et al. Variation across 141,456 human exomes and genomes reveals the spectrum of loss-offunction intolerance across human protein-coding genes. Available online: https://www.researchgate.net/publication/330708684_Variation_across_141456_human_exomes_and_genomes_reveals_the_spectrum_of_loss-of-function_intolerance_across_human_protein-coding_genes_Supplementary_Information/link/5e1822214585159aa4c2f45f/download (accessed on 21 February 2020).
14. Richards, S.; Aziz, N.; Bale, S.; Bick, D.; Das, S.; Gastier-Foster, J.; Grody, W.W.; Hegde, M.; Lyon, E.; Spector, E.; et al. Standards and guidelines for the interpretation of sequence variants: a joint consensus recommendation of the American College of Medical Genetics and Genomics and the Association for Molecular Pathology. *Genet. Med.* **2015**, *17*, 405-424, doi:10.1038/gim.2015.30.
15. Li, M.M.; Datto, M.; Duncavage, E.J.; Kulkarni, S.; Lindeman, N.I.; Roy, S.; Tsimberidou, A.M.; Vnencak-Jones, C.L.; Wolff, D.J.; Younes, A.; et al. Standards and guidelines for the interpretation and reporting of sequence variants in cancer: a Joint Consensus Recommendation of the Association for Molecular Pathology, American Society of Clinical Oncology, and College of American Pathologists. *J. Mol. Diagn.* **2017**, *19*, 4-23, doi:10.1016/j.jmoldx.2016.10.002.
16. Mayrhofer, M.; De Laere, B.; Whitington, T.; Van Oyen, P.; Ghysel, C.; Ampe, J.; Ost, P.; Demey, W.; Hoekx, L.; Schrijvers, D.; et al. Cell-free DNA profiling of metastatic prostate cancer reveals microsatellite instability, structural rearrangements and clonal hematopoiesis. *Genome Med.* **2018**, *10*, 85, doi:10.1186/s13073-018-0595-5.
17. Adalsteinsson, V.A.; Ha, G.; Freeman, S.S.; Choudhury, A.D.; Stover, D.G.; Parsons, H.A.; Gydush, G.; Reed, S.C.; Rotem, D.; Rhoades, J.; et al. Scalable whole-exome sequencing of cell-free DNA reveals high concordance with metastatic tumors. *Nat Commun* **2017**, *8*, 1324, doi:10.1038/s41467-017-00965-y.
18. Ha, G.; Roth, A.; Lai, D.; Bashashati, A.; Ding, J.; Goya, R.; Giuliany, R.; Rosner, J.; Oloumi, A.; Shumansky, K.; et al. Integrative analysis of genome-wide loss of heterozygosity and monoallelic expression at nucleotide resolution reveals disrupted pathways in triple-negative breast cancer. *Genome Res.* **2012**, *22*, 1995-2007, doi:10.1101/gr.137570.112.
19. RDocumentation. heatmap.2. Available online: https://www.rdocumentation.org/packages/gplots/versions/3.0.1.1/topics/heatmap.2 (accessed on 21 February 2020).
20. RDocumentation. dist - distance matrix computation. Available online: https://www.rdocumentation.org/packages/stats/versions/3.6.2/topics/dist (accessed on 21 February 2020).
21. McKenna, A.; Hanna, M.; Banks, E.; Sivachenko, A.; Cibulskis, K.; Kernytsky, A.; Garimella, K.; Altshuler, D.; Gabriel, S.; Daly, M.; et al. The Genome Analysis Toolkit: a MapReduce framework for analyzing next-generation DNA sequencing data. *Genome Res.* **2010**, *20*, 1297-1303, doi:10.1101/gr.107524.110.
22. Cui, Y.; Chen, X. BioCircos.R. Available online: https://github.com/lvulliard/BioCircos.R (accessed on 21 February 2020).

23. Steensma, D.P.; Bejar, R.; Jaiswal, S.; Lindsley, R.C.; Sekeres, M.A.; Hasserjian, R.P.; Ebert, B.L. Clonal hematopoiesis of indeterminate potential and its distinction from myelodysplastic syndromes. *Blood* **2015**, *126*, 9-16, doi:10.1182/blood-2015-03-631747.
24. Gibson, C.J.; Steensma, D.P. New insights from studies of clonal hematopoiesis. *Clin. Cancer Res.* **2018**, *24*, 4633-4642, doi:10.1158/1078-0432.CCR-17-3044.
25. Hale, V.; Weischer, M.; Park, J.Y. CHEK2*1100delC mutation and risk of prostate cancer. *Prostate Cancer* **2014**, *2014*, 294575, doi:10.1155/2014/294575.
26. Hallamies, S.; Pelttari, L.M.; Poikonen-Saksela, P.; Jekunen, A.; Jukkola-Vuorinen, A.; Auvinen, P.; Blomqvist, C.; Aittomaki, K.; Mattson, J.; Nevanlinna, H. CHEK2 c.1100delC mutation is associated with an increased risk for male breast cancer in Finnish patient population. *BMC Cancer* **2017**, *17*, 620, doi:10.1186/s12885-017-3631-8.
27. Wu, Y.; Yu, H.; Zheng, S.L.; Na, R.; Mamawala, M.; Landis, T.; Wiley, K.; Petkewicz, J.; Shah, S.; Shi, Z.; et al. A comprehensive evaluation of CHEK2 germline mutations in men with prostate cancer. *Prostate* **2018**, *78*, 607-615, doi:10.1002/pros.23505.
28. Apostolou, P.; Papasotiriou, I. Current perspectives on CHEK2 mutations in breast cancer. *Breast Cancer (Dove Med Press)* **2017**, *9*, 331-335, doi:10.2147/BCTT.S111394.
29. Kilpivaara, O.; Vahteristo, P.; Falck, J.; Syrjakoski, K.; Eerola, H.; Easton, D.; Bartkova, J.; Lukas, J.; Heikkila, P.; Aittomaki, K.; et al. CHEK2 variant I157T may be associated with increased breast cancer risk. *Int. J. Cancer* **2004**, *111*, 543-547, doi:10.1002/ijc.20299.
30. Wang, Y.; Dai, B.; Ye, D. CHEK2 mutation and risk of prostate cancer: a systematic review and meta-analysis. *Int. J. Clin. Exp. Med.* **2015**, *8*, 15708-15715.
